# Effect of food swap recommendations on the healthfulness and carbon footprint of grocery purchases in a naturalistic online store: A randomized parallel trial

**DOI:** 10.1371/journal.pmed.1004847

**Published:** 2026-08-21

**Authors:** Anna H. Grummon, Kevin O’Sullivan, Amanda B. Zeitlin, Lindsey Smith Taillie, Cristina J. Y. Lee, Ashe J. Walker, Verena Tiefenbeck, Elgar Fleisch, Clayton Ulm, Thomas N. Robinson

**Affiliations:** 1 Department of Pediatrics, Stanford University School of Medicine, Palo Alto, California, United States of America; 2 Department of Health Policy, Stanford University School of Medicine, Stanford, California, United States of America; 3 Department of Management, Technology, and Economics, ETH Zurich, Zurich, Switzerland; 4 Department of Nutrition, Gillings School of Global Public Health, University of North Carolina, Chapel Hill, North Carolina, United States of America; 5 Carolina Population Center, University of North Carolina, Chapel Hill, North Carolina, United States of America; 6 Center for Community Engagement, West Virginia University, Morgantown, West Virginia, United States of America; 7 School of Business, Economics and Society, Friedrich-Alexander-Universität, Nürnberg, Germany; 8 Institute of Technology Management, University of St. Gallen, St. Gallen, Switzerland; 9 Department of Health Behavior, Gillings School of Global Public Health, University of North Carolina, Chapel Hill, North Carolina, United States of America; 10 Department of Medicine, Stanford University School of Medicine, Palo Alto, California, United States of America; University of Cambridge, UNITED KINGDOM OF GREAT BRITAIN AND NORTHERN IRELAND

## Abstract

**Background:**

Modeling studies suggest that simple dietary “swaps” in which just one food is replaced with another could yield both health and environmental benefits, but it remains unclear whether consumers make such changes when prompted. We tested whether recommending swaps improved the healthfulness and reduced the carbon footprint of simulated online grocery selections.

**Methods and findings:**

In May–June 2025, we recruited 1,201 US adults ages 18+ for a randomized parallel trial. Participants completed 3 weekly study visits. In the first visit, participants shopped in a naturalistic online grocery store with no intervention. They were then randomized in parallel to 1 of 4 trial arms: (1) climate swaps (*n* = 301), (2) health swaps (*n* = 300), (3) climate + health swaps (*n* = 300), or (4) control (*n* = 300). In the second and third visits, participants completed the shopping task in their randomly assigned trial arm. Participants were not masked to their trial arm. Using intent-to-treat analyses, we assessed 2 primary endpoints: the healthfulness (operationalized as the average Ofcom Nutrient Profiling Model score across the selected items [range: 1–100]) and carbon footprint (operationalized as the average kg of carbon dioxide equivalents [CO_2_-eq] per kg associated with producing the selected items) of participants’ grocery selections. We examined whether outcomes changed from the first (no-intervention) visit to the second and third (intervention) visits in the swaps arms compared to the control arm.

The climate (*B* = 2.4 on the 1–100 scale, 95% CI [1.1, 3.7]), health (*B* = 4.7, 95% CI [3.3, 6.0]), and climate + health (*B* = 4.0, 95% CI [2.7, 5.4]) swaps each improved the healthfulness of participants’ selections compared to control (all *p*s < 0.001). The health and climate + health swaps both increased healthfulness more than the climate swaps (*p*s ≤ 0.02) but did not differ from one another (*p* = 0.34). The climate (*B* = −3.3 kg CO_2_-eq per kg, 95% CI [−4.6, −2.1]; *p* < 0.001) and climate + health swaps (*B* = −2.8, 95% CI [−4.1, −1.6]; *p* < 0.001), but not the health swaps (*B* = −0.4, 95% CI [−1.7, 0.8]; *p* = 0.51), reduced the carbon footprint of participants’ selections compared to control. The climate and climate + health swaps led to larger reductions in carbon footprint than the health swaps (*p*s < 0.001) but did not differ from one another (*p* = 0.43). Swaps’ effects on healthfulness and carbon footprint did not differ by age, health consciousness, or environmental consciousness (*p*s for interactions ≥0.10). The main limitations were that the sample differed somewhat from the US population and study results may not generalize to brick-and-mortar stores.

**Conclusions:**

Encouraging swaps to more healthful and lower-carbon foods improved the healthfulness and reduced the carbon footprint of online grocery selections.

**Clinical trials registration:**

ClinicalTrials.gov (NCT#06648226), first submitted October 16, 2024.

## Author summary

**Why was this study done?:**

- Modeling studies show that simple dietary substitutions (i.e., “swaps”) in which consumers replace one food with another similar food can improve the healthfulness of consumers’ diets and reduce the climate impact of food production.
- Retailers, including online grocery stores, have begun recommending health- or climate-focused food swaps, but little is known about how consumers respond to these swap recommendations in online grocery store settings.

**What did the researchers do and find?:**

- This study used a randomized parallel trial design in a realistic online grocery store to evaluate the effects of recommending swaps to lower-climate-impact foods (“climate swaps”), more healthful foods (“health swaps”), or both (“climate + health swaps”).
- Participants received an average of 2.3–3.6 swap recommendations and accepted an average of 25%–27% of these swaps.
- The climate, health, and climate + health swaps all improved the healthfulness of participants’ grocery selections, but only the climate and climate + health swaps reduced the climate impact of selections.

**What do these findings mean?:**

- Recommending swaps to more healthful or lower-climate-impact foods is a promising, scalable strategy for improving the healthfulness of online grocery purchases.
- Recommending swaps to lower-climate-impact foods may have the additional benefit of also reducing the climate impact of food production.
- The main limitations of the trial were that the sample differed somewhat from the US population overall and that study results may not generalize to brick-and-mortar stores.

## Abstract 3

In this randomized parallel trial, Anna Grummon and colleagues examine whether simple food-swap suggestions can influence online grocery choices. They show that recommendations focused on health, climate impact, or both were associated with healthier selections, while climate-focused and combined recommendations were also linked to lower-carbon grocery baskets in a simulated online shopping environment.

## Introduction

Changing the foods we eat could improve health and mitigate climate change. Poor diet quality accounts for one in five deaths globally [1], and food production accounts for up to one-third of human-caused greenhouse gas emissions [2]. Experts agree that substantial dietary change is needed to reduce both diet-related disease and greenhouse gas emissions [3,4].

Many consumers report wanting to eat healthier, more environmentally sustainable diets [5–9]. However, consumers often struggle to realize these goals [10,11], in part because many find it hard to understand which foods promote health or have lower climate impacts (e.g., emit fewer greenhouse gases) [8,9]. Dietary guidelines, academic reports, and nongovernmental organizations recommend diets high in fruits, vegetables, and legumes and low in red and processed meats [3,12–17]. However, these broad recommendations may be challenging to implement given the complexity of food choice, including the need to consider multiple dimensions of food and compare many choices simultaneously [18–20].

One approach to addressing these challenges is to encourage consumers to make simple dietary substitutions, or “swaps,” in which they replace one food with another similar food [21]. Swaps could improve health and lower the climate impact of food production because foods that are otherwise similar (e.g., a beef burger and a turkey burger) can differ substantially in both their healthfulness and in the carbon emissions released when they are produced (i.e., their “carbon footprint”) [21,22]. Swaps may also be easier to implement than more extensive dietary changes like eliminating meat [23–25].

Technological advances have made it possible to recommend swaps to consumers in real time as they decide what foods to buy. For example, Kroger—the largest grocer in the US [26]—offers an online program called “OptUP” that recommends swaps from less healthful foods to similar, more healthful foods [27] and apps like “ecoSwitch” allow consumers to scan foods’ barcodes to receive swap recommendations to similar, lower-climate-impact foods [28,29]. Consumers are increasingly interested in receiving these recommendations [30].

Although simulation studies highlight the potential for swaps to improve the healthfulness or reduce the climate impact of food purchases [21,22,31–33], little is known about how consumers respond to swap recommendations. A small body of research indicates that recommending more healthful food swaps can improve the healthfulness of food purchases (e.g., reduce sodium purchases) [34–37], but studies have not tested whether recommending lower-climate-impact food swaps reduces the carbon footprint of purchases. Also unknown is whether recommending more healthful swaps influences carbon footprints and vice versa. Health- and climate-focused swaps might generate positive spillovers on the other dimension (i.e., a win-win), given the overlap between more healthful and lower-climate-impact dietary patterns [3,12,38], or negative spillovers, because some more healthful foods are relatively high-climate-impact (e.g., low-fat dairy products) and some lower-climate-impact foods are relatively unhealthful (e.g., meat mimics) [39]. Given these potential tradeoffs, swap recommendation systems may need to address both dimensions simultaneously, but the effects of doing so remain unknown.

To address these gaps, we tested whether recommending more healthful swaps, lower-climate-impact swaps, or both improves the healthfulness and reduces the carbon footprint of grocery purchases. We conducted a randomized trial in a naturalistic online grocery store. Food and beverage purchases in naturalistic stores have been shown to correlate with purchases from real stores [40,41].

## Methods

### Study design and participants

The study adopted a randomized parallel group design. In 2025, we recruited a convenience sample of adults through the survey research panel Prolific. The first participant was enrolled on May 7, 2025, and the last on June 4, 2025; follow-up was completed on June 25, 2025. Inclusion criteria were being 18 years of age or older, residing in the US, being able to complete a survey in English, and having internet access to complete 3 online study visits; exclusion criteria were being younger than 18, residing outside of the US, being unable to complete a survey in English, or not having internet access to complete 3 online survey visits. We used quota sampling to ensure that approximately 50% of participants were young adults ages 18–25 to enable moderation analyses by age group, given that age may influence intervention receptivity [42,43]. We followed CONSORT-2025 guidelines for reporting clinical trials [44] (S1 Checklist). Participants were not involved in the design, conduct, or reporting of the trial. The study was prospectively registered (ClinicalTrials.gov NCT#06648226). There were no deviations from the protocol.

#### Ethics.

Participants provided electronic written informed consent. The Stanford University Institutional Review Board approved the study (IRB#76925). There were no amendments to the IRB during the course of the trial.

#### Setting.

The trial took place in a naturalistic online grocery store developed for this study. The store displayed 5,550 products across 6 departments (S1 Table); to ensure the store closely mirrored the look and feel of a real online grocer, product images, names, prices, and nutritional information were displayed as they were shown on a large US grocery chain’s website in September 2023. The store’s functionalities also mirrored a real online store, including browsing, search, product pages, sorting and filtering, and checkout [40]. Online stores like this one are realistic, acceptable, and valid settings for evaluating nutrition interventions [40,45].

#### Carbon footprint of products.

We assessed the climate impact of all products in the store, operationalized as products’ carbon footprint (i.e., the greenhouse gas emissions associated with producing the products). We focused on carbon footprints because manufacturers and retailers including Unilever and Walmart have pledged to reduce carbon footprints, including from downstream consumption [46–50]. We obtained information on carbon footprints from GreenChoice, which calculated kg of carbon dioxide equivalents (CO_2_-eq) per kg for each item using a comprehensive review of farm-to-gate life cycle analyses [51].

#### Healthfulness of products.

We assessed product healthfulness using Ofcom Nutrient Profiling Model scores [52,53]. The Ofcom system was developed by the United Kingdom to regulate food marketing to children; scores are associated with risk of developing diet-related disease [54,55]. Products with more healthful Ofcom scores generally had lower carbon footprints, although the strength of this association varied across food groups (S1 Table).

### Randomization and masking

**Fig 1:**
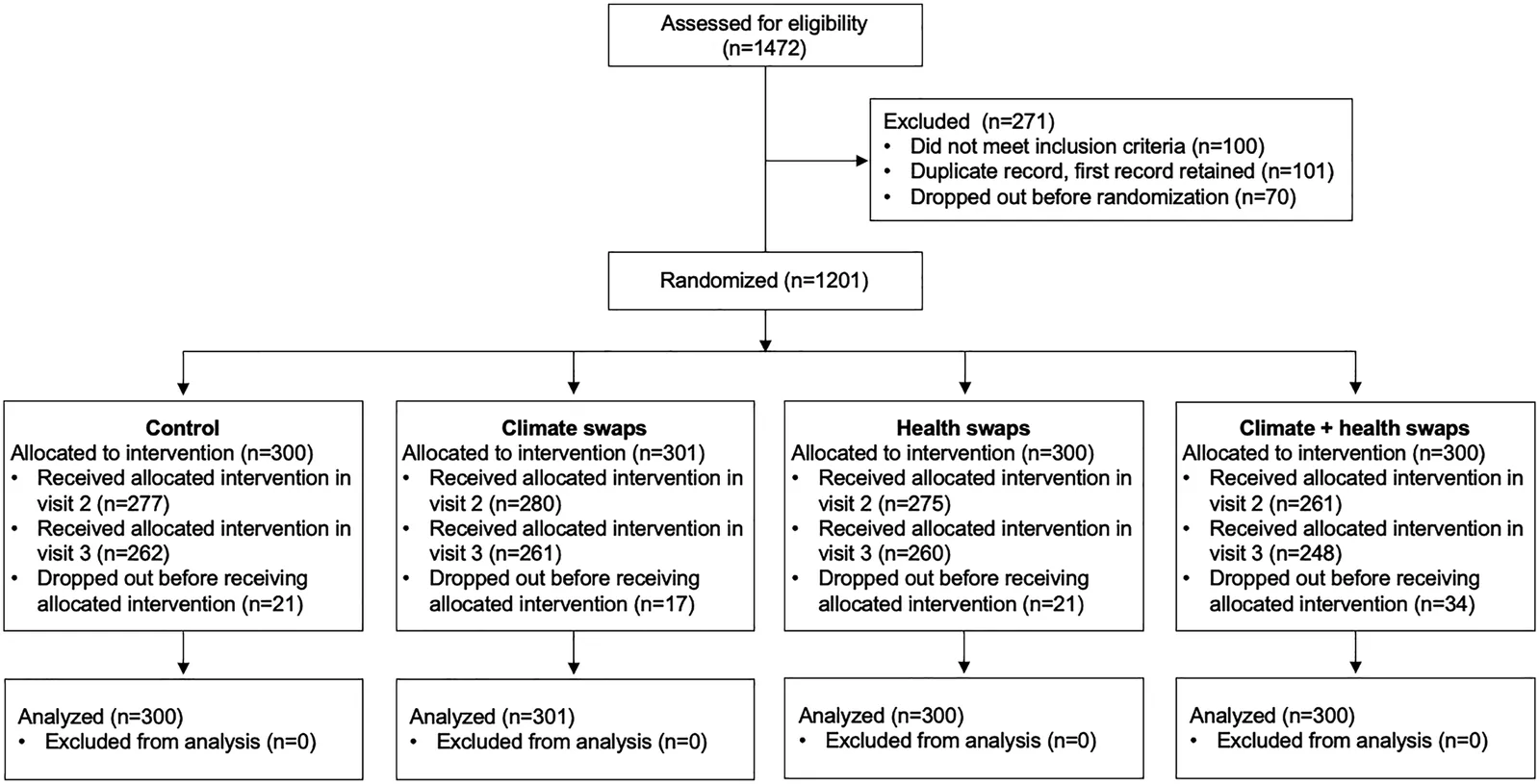
CONSORT flow diagram.

Participants completed 3 online study visits. Participants were randomized at the end of the first study visit by Qualtrics to 1 of 4 trial arms in a 1:1:1:1 allocation ratio: climate swaps, health swaps, climate + health swaps, or control (Fig 1). Interventions are described below. Participants were not masked to assignments.

### Procedures

Participants completed 3 online study visits, each approximately 1 week apart. At each visit, participants shopped in the naturalistic online store, then answered survey questions. In the first visit (baseline), participants provided electronic informed consent and shopped in the store with no intervention. Participants were randomized at the end of the first visit. In both the second and third visits, participants shopped in the store in their assigned arm; the third visit provided a second intervention exposure to enable us to test whether intervention effects changed with repeated exposure. At the start of each visit, participants completed a “commitment check” to encourage thoughtful responses [56].

When shopping in the store, participants were instructed to shop as they usually would for items in 6 food groups: beverages; boxed and frozen meals; milk and dairy products; meat and meat alternatives; soups; and sweets and snacks. These groups are top contributors to saturated fat, added sugar, sodium, and energy intake [57–62] or to the dietary carbon footprint in the US [21]. Participants were given a $50 budget based on national spending data [63] and 2022 sales data from the grocery chain on which the store was modeled. Participants could spend between 0.5 and 1.5 times this budget ($25 to $75) and otherwise shop freely. Participants were informed that 25 of them would be randomly chosen to receive the groceries selected in 1 of their visits as well as the remaining balance of their budget. This procedure incentivized participants to behave as they would in the real world, including selecting only items they actually wished to receive and considering whether they would rather have a particular item or retain the value of the item as cash. Participants were required to demonstrate understanding of this procedure before continuing. Because delivering groceries to a national sample of participants posed logistical challenges, we debriefed participants at the end of the study that those selected for delivery would instead receive an additional $50 incentive. Participants received $4.50, $6.00, and $8.00 USD for completing the first, second, and third visits, respectively.

#### Interventions.

**Fig 2:**
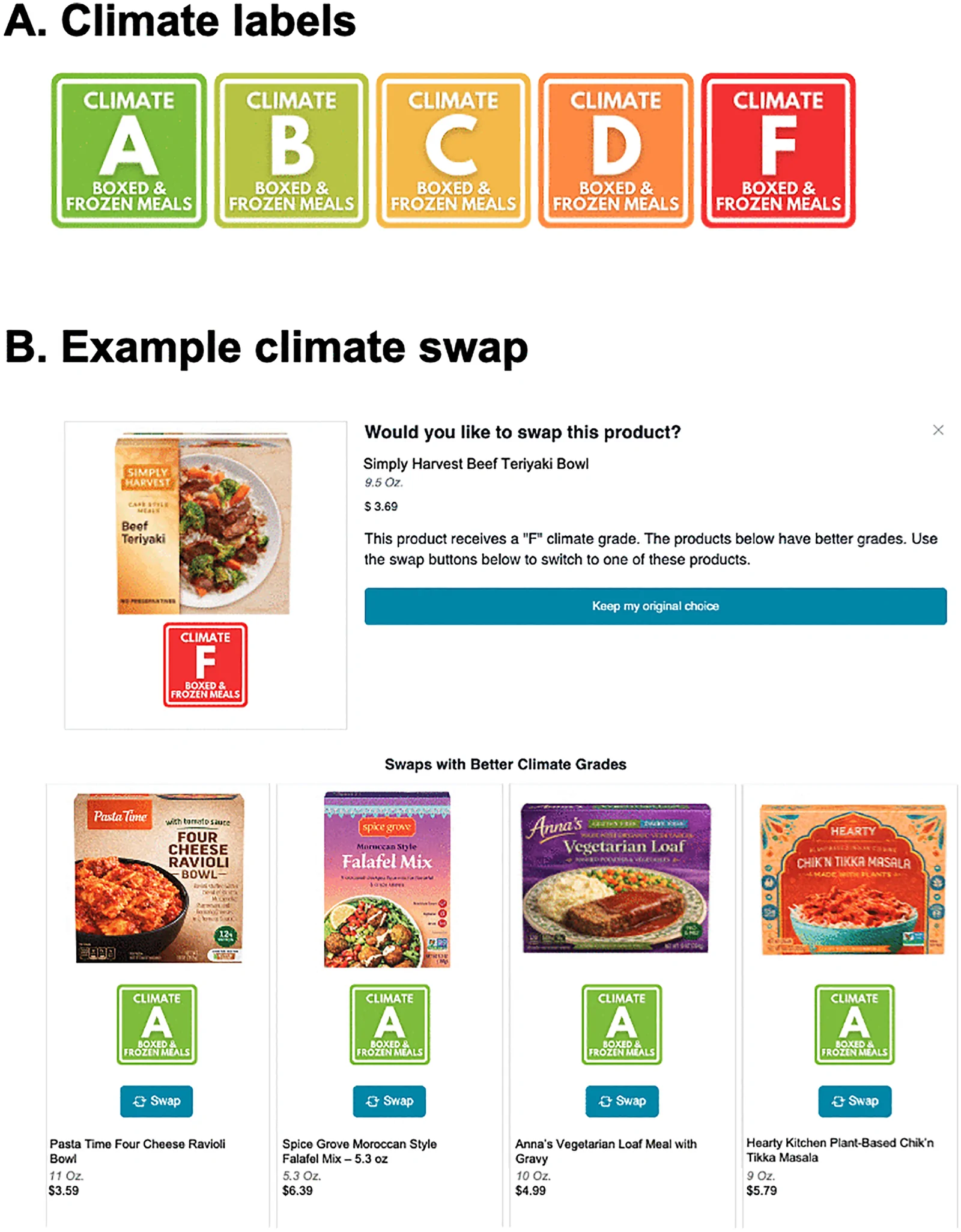
Example (A) climate labels and (B) climate swap. *Note*: Figure shows climate labels and an example climate swap. The health labels had the same design as the climate labels but used the word “Nutrition” instead of “Climate.” Panel B shows mock products to preserve copyright; participants saw real, branded products in the trial.

In the climate swaps arm, the grocery store displayed color-coded climate grade labels (“A,” “B,” “C,” “D,” and “F”) next to all products, based on quintiles of carbon footprints in each food group (S1 Text). Grades were selected to match the predominant US grading system, which does not use the letter “E.” The labels mimicked Nutri-Score labels [64–66] (Fig 2A). When participants attempted to add a product with a “C,” “D,” or “F” grade, the store automatically offered them swaps to lower-climate-impact products (“A” or “B” grade) within the same product subcategory, category, or department as the originally selected product (see Fig 2B for an example swap and S2 Table for the hierarchy of subcategories, categories, and departments). S1 Text details how swaps were identified and presented; briefly, the store identified all possible swaps (i.e., products with an “A” or “B” grade in the same subcategory, category, or department as the originally selected product) and presented an initial set of up to 8 products, beginning with those in the same subcategory as the originally selected product. Participants could load additional swaps, in batches of up to 8, until all possible swaps were shown. Climate swap recommendations were based solely on carbon footprint.

In the health swaps arm, the online grocery store displayed health grade labels based on Ofcom scores [52,53], using the same label design as above. We applied “A” or “B” grades to products above the United Kingdom’s regulatory cutoffs for marketing products to children [53] and “C,” “D,” and “F” grades to products below these cutoffs (S1 Text). When participants attempted to add a product with a “C,” “D,” or “F” grade, the store automatically offered swaps to similar but more healthful products (with an “A” or “B” grade) that were in the same product subcategory, category, or department as the originally selected product (S1 Text). Health swap recommendations were based solely on healthfulness.

In the climate + health swaps arm, the store displayed both the climate and health labels side-by-side next to all products. When participants attempted to add a product with a “C,” “D,” or “F” grade on either dimension, the store offered swaps that improved at least 1 dimension (carbon footprint or healthfulness) and were at least as good or better on the other, while never recommending products with a “C,” “D,” or “F” grade on either dimension (S1 Text).

### Outcomes

The 2 prespecified primary endpoints were the healthfulness and carbon footprint of participants’ selections. Healthfulness was defined as the average Ofcom score of selected products, converted to a 1–100 scale (higher scores indicate more healthful selections) [53]. Carbon footprint was defined as the mean greenhouse gas emissions intensity (i.e., kg of carbon dioxide equivalents [CO_2_-eq] per kg) of selected products. The study evaluated several secondary endpoints, including both selection endpoints (related to participants’ grocery selections) and psychological endpoints (assessed via survey). Secondary selection endpoints were energy (i.e., calorie) and nutrient (i.e., sugar, sodium, saturated fat, fiber, and protein) density of selections and total spending. Secondary psychological outcomes were selected based on health behavior theory and previous research [67–73]. These outcomes were thinking about health, climate impact, and taste while shopping (also called “cognitive elaboration”); feeling negative (i.e., guilt, shame, worry) and positive (i.e., inspiration, pride, reassurance) emotions while shopping; and injunctive and descriptive norms (i.e., beliefs about what people should do and what they typically do, respectively) about buying healthy foods and foods with low climate impact. As an exploratory endpoint, the survey also assessed acceptability of the labels and swaps [74].

Finally, the survey assessed participants’ characteristics, including demographics, health consciousness [75,76], and environmental consciousness [77], and their reactions to the store (S3 Table shows all measures). All outcomes were pre-registered prior to data collection and are presented in the current manuscript. Harms were assessed by participant report.

### Statistical analysis

We pre-registered the analysis plan (ClinicalTrials.gov NCT#06648226; S1 Protocol); deviations to this plan are noted. Analyses were intent-to-treat and used a two-tailed alpha = 0.05. We conducted analyses in Stata MP version 19.

First, we summarized the number of swaps offered and accepted. Second, we used linear mixed models to estimate the effects of the 3 swaps interventions on the 2 primary outcomes, pooling across the first and second exposure to maximize power to detect effects (S1 Text). Mixed effects models handled missing data using maximum likelihood. To estimate within-person change from baseline, we regressed the outcome on indicator variables for each trial arm (i.e., climate swaps, health swaps, and climate + health swaps, treating the control group as the reference category; this adjusts for baseline values of the outcome), study visit (treating the baseline visit as the reference category), the interaction between trial arm and visit, and a random intercept to account for repeated measures within participants. We did not include any covariates given the randomized design. We used the models to estimate changes from baseline to the intervention period (weighted average of visits 2 and 3) relative to control and to test differences between the interventions. We estimated corrected *p*-values using the Bonferroni-Holm method [78], considering 6 tests for the primary outcomes (each intervention arm versus control for the 2 primary outcomes) and 3 tests for the secondary outcomes (each intervention arm versus control); these corrections were not pre-registered. This change did not affect the statistical significance of the results overall, except for two tests examining thinking about health. We converted effect sizes to standardized effects (Cohen’s *d*) using the Campbell Collaboration’s Effect Size Calculator [79], interpreting effects of *d* = .20 as small, .50 as medium, and .80 as large [80].

Third, we examined intervention effects on the primary outcomes within food groups (e.g., separately for beverages, meals, etc.); these analyses were exploratory and not pre-registered. Fourth, we examined whether the effects of the swaps interventions on the 2 primary outcomes were moderated by age (young adult [18–25 years] versus older adult [≥26 years]), health consciousness (treated continuously), and environmental consciousness (treated continuously) by adding indicator variables for the moderator and interactions between the moderator, trial arm, and study visit to the main model. We used separate models for each moderator and tested the joint significance of the three-way interaction terms. We chose these moderators because they may influence receptivity to the interventions [42,43,77,81]. Fifth, we examined whether intervention effects on the 2 primary outcomes changed from the first to the second exposure by examining the contrast between effects at the first versus second exposure. Sixth, we examined effects on secondary outcomes using the same mixed effects approach. Seventh, we described intervention acceptability (e.g., proportion who liked the swaps).

We estimated *a priori* sample size needs to detect an effect of each intervention versus control of *d* = .15 or larger, a small effect [80] that is conservative based on prior studies [34,82,83]. *A priori* power calculations assumed a two-tailed alpha = 0.05 and within-person correlation = .40, and 90% power. Under these assumptions, a sample of 904 (226 per group) would yield 90% power to detect an effect of this size or larger. To allow for missing data, we aimed to recruit 1,200 participants (300 per group). We ultimately included 1,201 participants in analyses; assuming a more conservative alpha of 0.008 (0.05 ÷ 6 tests; more conservative than the Bonferroni-Holm correction we applied *post hoc*), this achieved sample size yielded 87% power to detect effects of *d* = .15 or larger.

### Artificial intelligence tools and technologies

We used ChatGPT 4o-mini to create the mock product images shown in Fig 2B. We show mock products in the figure to preserve copyright; participants saw real, branded products in the trial. No other content in the manuscript or supporting files was generated by artificial intelligence tools or technologies.

## Results

### Sample characteristics

A total of 1,201 participants were randomized and included in analyses (Fig 1). No adverse events were reported. Participants’ mean [SD] age was 31.5 [12.2] years and 53% (638/1,201) identified as women (Table 1). About two-thirds (67%; 810/1,201) reported annual household income <$100,000. Compared with the US population, participants were more likely to be young adults (consistent with recruitment goals), identify as Black or African American, have a college or graduate degree, and have income <$100,000, and were less likely to identify as Latino or Hispanic or multiracial (S4 Table).

**Table 1:** Participant characteristics, *n* = 1,201 US adults.

Characteristic	Participants, *N* (%)									
**Overall** ***n* = 1,201**		**Control** ***n* = 300**		**Climate swaps** ***n* = 301**		**Health swaps** ***n* = 300**		**Climate + health swaps** ***n* = 300**	
Age										
18–25 years	601	(50%)	156	(52%)	139	(46%)	148	(49%)	158	(53%)
26–39 years	344	(29%)	88	(29%)	96	(32%)	79	(26%)	81	(27%)
40–59 years	208	(17%)	44	(15%)	60	(20%)	57	(19%)	47	(16%)
60 years or older	48	(4%)	12	(4%)	6	(2%)	16	(5%)	14	(5%)
Gender										
Woman	638	(53%)	160	(53%)	157	(52%)	164	(55%)	157	(52%)
Man	545	(45%)	134	(45%)	138	(46%)	132	(44%)	141	(47%)
Non-binary or another gender	18	(1%)	6	(2%)	6	(2%)	4	(1%)	2	(0.7%)
Latino(a) or Hispanic Ethnicity	95	(8%)	23	(8%)	24	(8%)	27	(9%)	21	(7%)
Race										
American Indian or Alaska Native	7	(0.6%)	2	(0.7%)	2	(0.7%)	1	(0.3%)	2	(0.7%)
Asian, Native Hawaiian, or another Pacific Islander	56	(5%)	21	(7%)	11	(4%)	13	(4%)	11	(4%)
Black or African American	377	(31%)	100	(33%)	89	(30%)	92	(31%)	96	(32%)
White	713	(59%)	165	(55%)	187	(62%)	180	(60%)	181	(60%)
Another race or multiracial	48	(4%)	12	(4%)	12	(4%)	14	(5%)	10	(3%)
Education										
High school diploma or less	128	(11%)	30	(10%)	38	(13%)	34	(11%)	26	(9%)
Some college or associate’s degree	194	(16%)	60	(20%)	47	(16%)	40	(13%)	47	(16%)
College graduate	557	(46%)	129	(43%)	136	(45%)	145	(48%)	147	(49%)
Graduate degree	322	(27%)	81	(27%)	80	(27%)	81	(27%)	80	(27%)
Household income, annual										
$0 to $49,999	335	(28%)	78	(26%)	95	(32%)	77	(26%)	85	(28%)
$50,000 to $99,999	475	(40%)	128	(43%)	108	(36%)	121	(40%)	118	(39%)
$100,000 to $149,999	246	(20%)	63	(21%)	57	(19%)	62	(21%)	64	(21%)
$150,000 or more	145	(12%)	31	(10%)	41	(14%)	40	(13%)	33	(11%)
Household size										
1–2	373	(31%)	87	(29%)	94	(31%)	100	(33%)	92	(31%)
3–4	578	(48%)	151	(50%)	141	(47%)	129	(43%)	157	(52%)
5 or more	248	(21%)	62	(21%)	64	(21%)	71	(24%)	51	(17%)

*Note.* Missing data ranged from 0.0% to 0.7% across arms.

### Acceptability of the online store

Participants found the online store realistic and acceptable (S1 Fig). For example, 94% (1,133/1,201) reported they would pick similar products in the real world as they selected in the online store.

### Swaps offered and accepted

Participants selected a mean [SD] of 11.3 items [4.0]. Pooling across intervention visits, participants in the climate swaps arm were offered a mean [SD] of 2.4 [2.8] swaps, those in the health swaps arm 2.3 [2.7] swaps, and those in the climate + health swaps arm 3.6 [3.8] swaps. On average, participants in the climate (25%), health (27%), and climate + health (27%) swaps arms accepted a similar proportion of the swaps offered.

### Healthfulness of grocery selections

**Fig 3:**
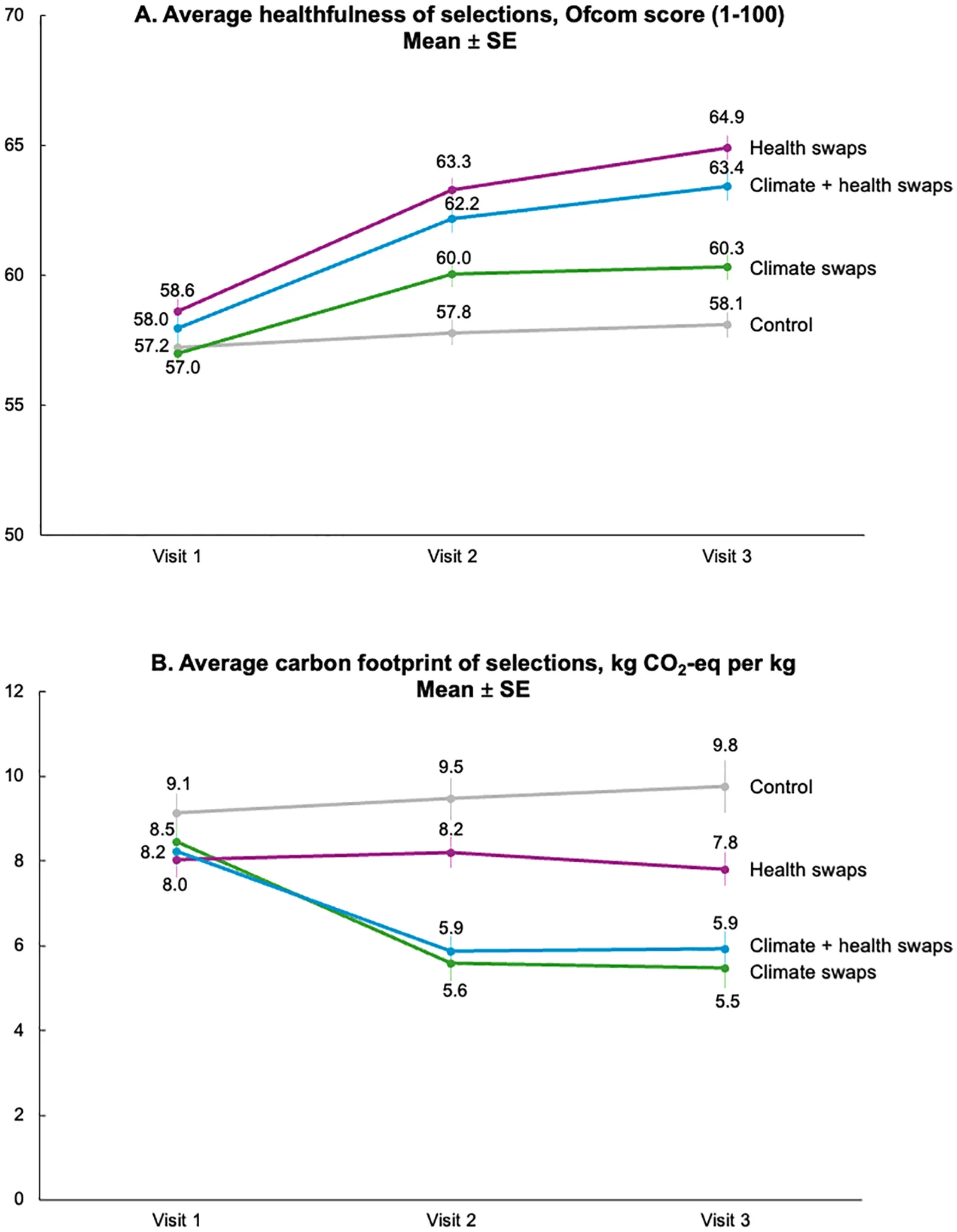
Effects of the climate, health, and climate + health swaps on (A) healthfulness and (B) carbon footprint of grocery selections by study visit. *Note:* Figure shows raw means and standard errors (SEs).

At baseline (no intervention), participants selected groceries with a mean [SD] healthfulness of 57.7 [8.2] on the 1–100 scale. Compared with control, the climate (*B* = 2.4, 95% CI [1.1, 3.7]), health (*B* = 4.7, 95% CI [3.3, 6.0]), and climate + health (*B* = 4.0, 95% CI [2.7, 5.4]) swaps all improved the healthfulness (all corrected *p*s < 0.001, Table 2, Fig 3). The health (difference versus climate swaps = 2.3, 95% CI [0.9, 3.6]; *p* < 0.001) and climate + health (difference versus climate swaps = 1.6, 95% CI [0.3, 3.0]; *p* = 0.02) swaps both increased the overall healthfulness of selections more than climate swaps alone but did not differ from one another (*p* = 0.34). The benefits of the health and climate + health swaps on healthfulness were observed for most food groups, whereas the benefits of the climate swaps on healthfulness were observed for only two food groups, meat and meat alternatives and sweets and snacks (S5 Table). The effects of the swaps on healthfulness did not differ by age group (*p* for joint significance of interaction terms = 0.17), health consciousness (*p* = 0.97), or environmental consciousness (*p* = 0.41).

**Table 2:** Effects of the climate, health, and climate + health swaps on food and beverage purchases and psychological outcomes, *n* = 1,201 US adults.

Outcome	Climate swaps					Health swaps					Climate + health swaps				
Selection outcomes	*B*	(95% CI)	Uncorr. *p*-value	Corr. *p*-value	Cohen’s *d*	*B*	(95% CI)	Uncorr. *p*-value	Corr. *p*-value	Cohen’s *d*	*B*	(95% CI)	Uncorr. *p*-value	Corr. *p*-value	Cohen’s *d*
Healthfulness, Ofcom score (1–100)	**2.4**	**(1.1, 3.7)**	**<0.001**	**<0.001**	**0.25**	**4.7**	**(3.3, 6.0)**	**<0.001**	**<0.001**	**0.50**	**4.0**	**(2.7, 5.4)**	**<0.001**	**<0.001**	**0.43**
Carbon footprint, kg CO_2_-eq per kg	**−3.3**	**(−4.6, −2.1)**	**<0.001**	**<0.001**	**−0.42**	−0.4	(−1.7, 0.8)	0.51	0.51	−0.05	**−2.8**	**(−4.1, −1.6)**	**<0.001**	**<0.001**	**−0.35**
Energy density, kcal per 100 g	**−15.1**	**(−29.1, −1.0)**	**0.035**	**0.048**	**−0.16**	**−16.9**	**(−31.0, −2.8)**	**0.019**	**0.048**	**−0.18**	**−17.4**	**(−31.6, −3.3)**	**0.016**	**0.048**	**−0.18**
Sugar density, grams per 100 g	−0.6	(−1.7, 0.6)	0.31	0.31	−0.08	**−1.9**	**(−3.0, −0.7)**	**0.001**	**0.003**	**−0.26**	**−2.0**	**(−3.2, −0.9)**	**<0.001**	**0.002**	**−0.28**
Sodium density, milligrams per 100 g	−7.1	(−43.9, 29.8)	0.71	0.71	−0.03	−34.6	(−71.5, 2.3)	0.07	0.20	−0.15	−27.5	(−64.7, 9.7)	0.15	0.29	−0.12
Saturated fat density, grams per 100 g	**−0.8**	**(−1.1, −0.4)**	**<0.001**	**<0.001**	**−0.33**	**−0.8**	**(−1.2, −0.5)**	**<0.001**	**<0.001**	**−0.34**	**−1.0**	**(−1.3, −0.6)**	**<0.001**	**<0.001**	**−0.42**
Fiber density, grams per 100 g	0.0	(−0.2, 0.2)	0.87	0.87	0.01	**0.3**	**(0.1, 0.5)**	**0.003**	**0.010**	**0.25**	0.2	(−0.01, 0.4)	0.06	0.13	0.16
Protein density, grams per 100 g	−0.4	(−1.0, 0.2)	0.16	0.49	−0.10	0.3	(−0.2, 0.9)	0.27	0.55	0.08	0.0	(−0.6, 0.6)	0.96	0.96	0.00
Total spending, US dollars	**−2.3**	**(−3.4, −1.3)**	**<0.001**	**<0.001**	**−0.23**	**−1.7**	**(−2.7, −0.7)**	**0.001**	**0.002**	**−0.17**	**−1.4**	**(−2.4, −0.3)**	**0.009**	**0.009**	**−0.14**
**Psychological outcomes**															
Thinking about															
Health	0.15	(0.01, 0.29)	0.036	0.07	0.10	**0.17**	**(0.03, 0.31)**	**0.016**	**0.049**	**0.12**	0.15	(0.01, 0.29)	0.036	0.07	0.10
Climate impact	**0.85**	**(0.69, 1.01)**	**<0.001**	**<0.001**	**0.47**	0.02	(−0.14, 0.18)	0.85	0.85	0.01	**0.73**	**(0.56, 0.89)**	**<0.001**	**<0.001**	**0.40**
Taste	−0.02	(−0.14, 0.10)	0.74	>0.99	−0.02	−0.05	(−0.17, 0.07)	0.43	>0.99	−0.06	−0.06	(−0.18, 0.06)	0.34	>0.99	−0.07
Emotional reactions															
Negative emotions	**0.17**	**(0.07, 0.26)**	**<0.001**	**0.001**	**0.23**	**0.13**	**(0.04, 0.22)**	**0.006**	**0.006**	**0.18**	**0.17**	**(0.07, 0.26)**	**<0.001**	**0.001**	**0.23**
Positive emotions	0.02	(−0.10, 0.15)	0.71	>0.99	0.01	0.09	(−0.03, 0.22)	0.15	0.46	0.05	0.02	(−0.11, 0.15)	0.75	>0.99	0.01
Norms															
Injunctive norms to buy healthy foods	0.04	(−0.07, 0.14)	0.49	>0.99	0.04	0.04	(−0.06, 0.15)	0.41	>0.99	0.04	0.01	(−0.10, 0.11)	0.91	>0.99	0.01
Descriptive norms about buying healthy foods	−0.07	(−0.22, 0.08)	0.34	0.68	−0.05	−0.04	(−0.19, 0.11)	0.57	0.68	−0.03	−0.14	(−0.29, 0.02)	0.08	0.23	−0.09
Injunctive norms to buy low-climate-impact foods	0.07	(−0.08, 0.21)	0.35	>0.99	0.04	0.02	(−0.13, 0.16)	0.82	>0.99	0.01	0.05	(−0.10, 0.20)	0.50	>0.99	0.03
Descriptive norms about buying low-climate-impact foods	0.05	(−0.09, 0.20)	0.47	0.95	0.03	−0.07	(−0.22, 0.07)	0.31	0.94	−0.05	0.03	(−0.12, 0.17)	0.70	0.95	0.02

*Abbreviations:* CI, confidence interval; CO_2_-eq, carbon dioxide equivalents; corr. *p*-value, corrected *p-*value; uncorr. *p*-value, uncorrected *p*-value.

*Note:* Table shows the impact of each swaps intervention compared to control (no intervention), given as the difference in changes from baseline to follow-up between the swaps arm and the control arm (B) along with the *p*-value on this difference and this difference converted to a standardized effect size (Cohen’s *d*). Table shows effects pooled across the first and second exposure to the swaps. Estimates are from linear mixed models, regressing the outcome on indicator variables for each trial arm, study visit, the interaction between trial arm and visit, and a random intercept to account for repeated measures within participants. Table shows both uncorrected *p*-values and *p*-values corrected for multiple comparisons (considering 6 tests for the primary outcomes and 3 tests for the secondary and other outcomes) using the Bonferroni-Holm method. Some corrected *p*-values are identical to uncorrected values because the Bonferroni-Holm method is a sequential procedure; for the test ranked last in the sequence (the largest *p*-value), the correction factor is 1 [78]. Also note that the monotonicity constraint ensures that *p*-values are monotonically increasing, so any corrected *p*-value that calculates to less than its predecessor in the sequence is adjusted upward to match the preceding value [78]. **Bolded** effects are statistically significant at alpha = 0.05 using the corrected *p*-values.

The effects of the health and climate + health swaps on the healthfulness of food selections increased somewhat after a second exposure (visit 3 versus visit 2, differences = 1.3 and 1.2, respectively), but these differences were not statistically significant (*p*s = 0.11 and 0.13, respectively) (Fig 3). The effects of the climate swaps on healthfulness did not change across exposures (*p* = 0.94).

### Carbon footprint of grocery selections

At baseline, participants selected groceries with mean [SD] carbon footprint of 8.5 kg [7.1] CO_2_-eq per kg. Compared with control, the climate (*B* = −3.3, 95% CI [−4.6, −2.1]; corrected *p* < 0.001; approximate 39% reduction) and climate + health swaps (*B* = −2.8, 95% CI [−4.1, −1.6]; corrected *p* < 0.001; approximate 33% reduction), but not the health swaps (*B* = −0.4, 95% CI [−1.7, 0.8]; corrected *p* = 0.51), reduced the carbon footprint of participants’ selections. Both the climate (difference versus health swaps = −2.9, 95% CI [−4.2, −1.7]; *p* < 0.001) and climate + health (difference versus health swaps = −2.4, 95% CI [−3.7, −1.2]; *p* < 0.001) swaps produced larger reductions in carbon footprint overall than the health swaps but did not differ from one another (*p* = 0.43). The benefits of the climate swaps on carbon footprints were observed across all food groups except beverages, whereas the benefits of the climate + health swaps were observed only for meat and meat alternatives, soups, and sweets and snacks (S5 Table). Although the health swaps did not reduce carbon footprint overall, they reduced the carbon footprint of milk and dairy selections and sweets and snacks selections (S5 Table). The effects of the swaps on carbon footprint did not differ by age group (*p* for joint significance of interactions = 0.34), health consciousness (*p* = 0.10), or environmental consciousness (*p* = 0.35). Additionally, the effects of the swaps on the carbon footprint of participants’ grocery selections did not change across exposures (*p*s > .39, Fig 3).

### Energy and nutrient densities and total spending

All three swap interventions (climate, health, and climate + health) reduced the energy and saturated fat density of selections compared to control (corrected *p*s < 0.05; Table 2). The health and climate + health swaps also reduced the sugar density of selections. Only the health swaps increased the fiber density of selections, and none of the interventions affected sodium or protein density. All three interventions reduced total spending (corrected *p*s < 0.01).

### Psychological responses

The health swaps increased how much participants reported thinking about health while shopping (corrected *p*s < 0.05), but the climate and climate + health swaps did not (both corrected *p*s = 0.07) (Table 2). Both the climate and climate + health swaps (both corrected *p*s < 0.001), but not the health swaps, increased thinking about climate impact. None of the interventions affected thinking about taste. All three interventions increased negative emotions while shopping (all corrected *p*s ≤ 0.01), and none affected positive emotions, injunctive norms, or descriptive norms.

### Acceptability of the interventions

**Fig 4:**
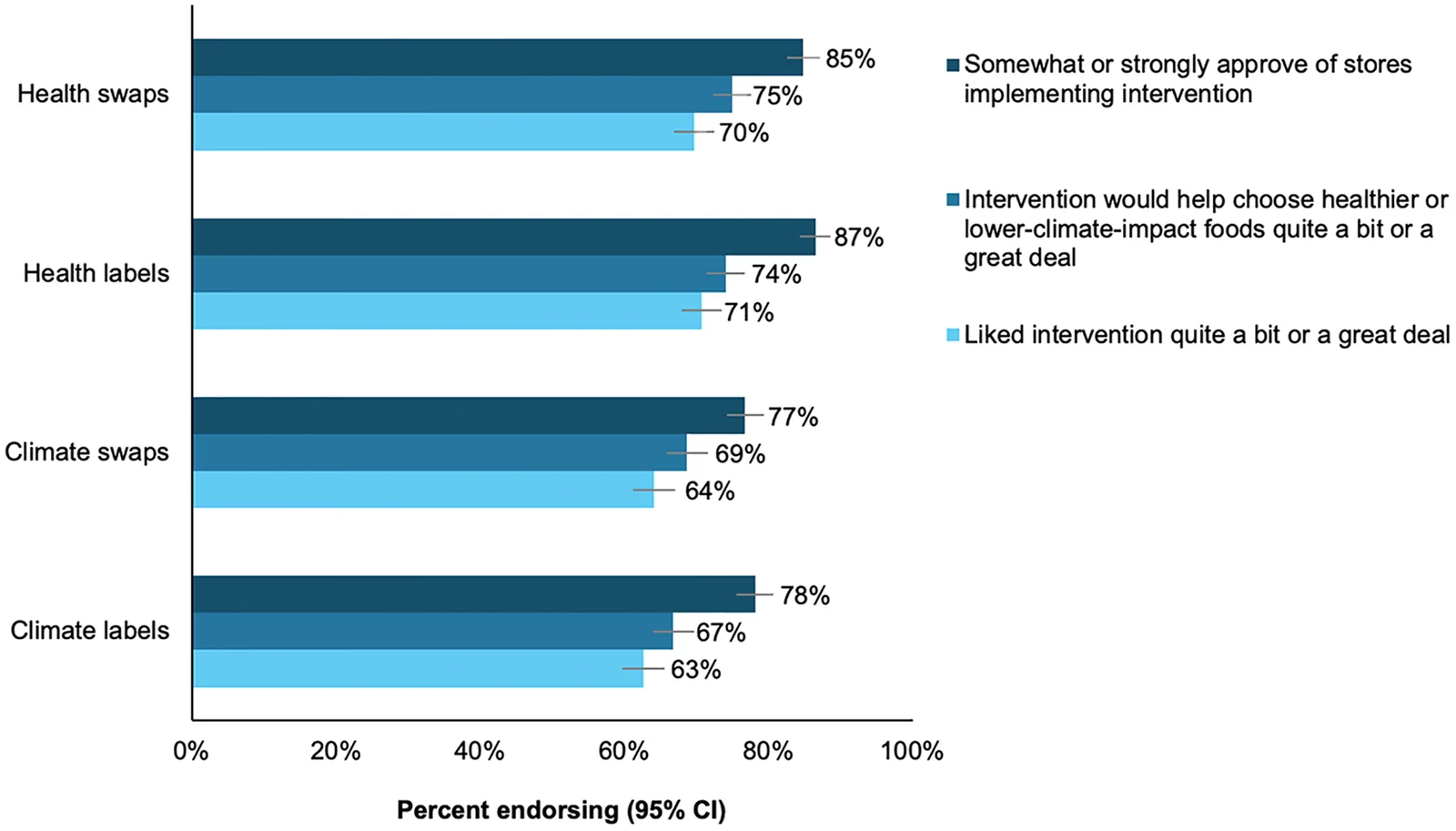
Acceptability of swaps and labels.

Participants perceived the interventions as acceptable. Across trial arms, a large majority of participants somewhat or strongly approved of grocery stores implementing the climate (77%, 791/1,030) and health (85%, 873/1,030) swaps and the accompanying climate (78%, 805/1,030) and health (87%, 891/1,030) labels. Most also indicated that the swaps and labels would help them quite a bit or a lot in choosing lower-climate-impact or healthier foods and that they liked the swaps and labels quite a bit or a lot (Fig 4).

## Discussion

In this randomized trial, climate and health swap recommendations, alone and in combination with one another, improved the healthfulness of grocery selections. Although improvements appear modest (approximately 2–5 points on the Ofcom scale), a prospective cohort study found that each 2-point increase in diet quality as assessed by Ofcom scores was associated with 14% lower risk of developing cardiovascular disease [55]. The climate swaps, alone or in combination with health swaps, also reduced the average carbon footprint of grocery selections by approximately 33%–39%, whereas the health swaps alone did not. Several major retailers already recommend swaps to their customers [27–29]; these findings suggest that such recommendations—whether focused on climate, health, or both—are a promising strategy to improve the healthfulness of grocery purchases and that climate-focused swaps may additionally reduce food-related greenhouse gas emissions.

The climate swaps improved the healthfulness of grocery selections even when offered without health swaps. By contrast, the health swaps alone did not reduce the carbon footprint of selections. The climate swaps also led participants to think more about health while shopping, while the health swaps did not increase thinking about climate impact. These results suggest asymmetric halo effects in which consumers generalize to perceive lower-climate-impact products to be more healthful, but do not generalize the other direction to perceive more healthful products as having lower climate impact. Although halo effects can sometimes undermine healthy or sustainable decision-making [84], the lower-climate-impact products offered in our store were, on average, more healthful, suggesting that generalizing was not unreasonable. Climate-focused interventions may therefore serve as a “stealth” strategy for encouraging more healthful purchases [85], particularly among consumers less motivated by health-focused approaches.

The health swaps improved the healthfulness of grocery selections more than the climate swaps, whereas the climate swaps reduced the carbon footprint of selections more than the health swaps. Combining the health and climate swaps did not dilute the effects of either alone: the climate + health swaps improved healthfulness to a similar extent as the health swaps alone and reduced carbon footprints to a similar extent as the climate swaps alone. These findings suggest that combining climate and health swaps does not confuse or overwhelm consumers or cause them to ignore both swaps or prioritize only one dimension (climate or health). Prior studies of combined climate and health labels have reported similar results [86,87]. Together, this evidence indicates that combining climate and health swaps may be a particularly promising strategy for addressing both diet-related disease and dietary carbon emissions. Moreover, both types of swaps were highly acceptable to participants.

The effects of the swaps did not vary by age group, health consciousness, or environmental consciousness. These results are consistent with studies of climate labels on restaurant menus [88,89] and taxes and warnings on red meat [73], which likewise found no differences in intervention effects by these characteristics. Together, this evidence suggests that swap recommendations may work equally well across a range of population groups.

Participants were exposed to the swaps twice, approximately 1 week apart. For participants in the health or climate + health swaps arms, we observed a modest but non-significant increase in healthfulness of selections after the second exposure compared to the first. This pattern is consistent with observational studies finding that the impact of graphic cigarette warnings may grow over time [90,91], though other research reports that warning effects attenuate over time [92]. By contrast, we observed no additional reductions in carbon footprint after the second exposure to the climate or climate + health swaps. Although longer studies will be needed, these results suggest that the benefits of health and climate swaps do not diminish rapidly and that the benefits of health swaps may increase modestly with repeated exposures.

Few studies have examined how swap recommendations exert their effects on behavior. In this study, the health swaps led consumers to think more about health while they were shopping (a process sometimes referred to as “cognitive elaboration”), and the climate swaps (alone or in combination with the health swaps) led consumers to think more about climate impact. All three interventions also increased negative emotions such as guilt and worry. Some scholars describe these negative emotional reactions as an “emotional tax” that should be weighed alongside intervention benefits [93,94], whereas others suggest that negative emotions can be an appropriate and meaningful response to certain interventions. Indeed, information that fails to elicit emotion often lacks meaning and is weighed less heavily in decision-making, potentially resulting in worse decisions [95–97]. Either way, our results suggest that cognitive elaboration and negative emotions may help explain how swap recommendations influence grocery purchases.

Participants in the intervention arms were offered approximately 2–4 swaps, indicating that only approximately 20%–30% of their original selections received “C,” “D,” or “F” grades. These results suggest that health and climate labels alone may have encouraged participants to select healthful or low-climate-impact items even before they were offered swaps. However, because labels and swaps were always delivered together in our study, we cannot disentangle their independent effects.

Strengths of this study included the use of a randomized controlled design, naturalistic online grocery store, and repeated exposures to the interventions. Limitations include using a convenience sample that differed somewhat from the overall US population, though a large body of evidence finds that trials in convenience samples typically produce similar results to representative samples [98–102]. Second, participants shopped in a simulated online grocery store (not an actual store) and did not pay for or receive their selections. However, they were incentivized to choose items they actually wanted, and prior research shows good correspondence between selections in simulated online grocery stores and real-world purchases [40]. Third, although the online store included more than 5,000 products, some food groups were not represented, so the effects on selections in those groups remain unknown. Fourth, we addressed price by having prices be the same across arms and designing the study to incentivize participants to consider cost as they would in the real world. However, we did not design swaps so that recommended products were similar in price to originally selected products. Swap acceptance may be higher when swaps are similarly priced to originally selected products; future studies could evaluate such price-neutral swaps. Fifth, we tested swaps in the context of an online grocery store, so effects in brick-and-mortar stores remain unknown. Sixth, we tested a combined climate + health intervention that presented both labels side-by-side and recommended swaps based on both labels. However, some retailers integrate climate and health information into a single score and display labels and swaps based on this integrated score; our results may not generalize to these systems. Seventh, data limitations precluded us from examining why participants accepted certain swaps and rejected others; future studies could explore what makes swaps acceptable to consumers, for example using qualitative approaches.

In summary, this randomized trial found that recommending swaps to more healthful or lower-climate-impact foods increases the healthfulness of grocery selections and recommending swaps to lower-climate-impact foods also reduces the carbon footprint of grocery selections. Combining both types of swaps improved both outcomes without diluting the impact of either intervention alone. Offering combined health- and climate-focused swaps in online grocery stores represents a promising, scalable strategy to reduce diet-related disease and the environmental impacts of food consumption.

## Supporting information

S1 Table Products shown in the naturalistic online store used in the trial. (DOCX)

S2 Table Departments, categories, and subcategories used in the naturalistic online store used in the trial. (DOCX)

S3 Table Survey measures used in trial. (DOCX)

S4 Table Comparison of characteristics of the study sample (*n* = 1,201 US adults) to national estimates. (DOCX)

S5 Table Effects of the climate, health, and climate + health swaps on the healthfulness and carbon footprint of food and beverage purchases, by food group, *n* = 1,201 US adults. (DOCX)

S1 Text Supplemental Methods. (DOCX)

S1 Fig Acceptability of the naturalistic online store used in the trial. (DOCX)

S1 Protocol Approved IRB Protocol and Statistical Analysis Plan. (PDF)

S1 Checklist CONSORT-2025 Checklist. Hopewell S, Chan AW, Collins GS, Hróbjartsson A, Moher D, Schulz KF, et al. CONSORT 2025 Statement: updated guideline for reporting randomised trials. BMJ. 2025; 388:e081123. https://dx.doi.org/10.1136/bmj-2024-081123. 2025 Hopewell and colleagues. This is an Open Access article distributed under the terms of the Creative Commons Attribution License (https://creativecommons.org/licenses/by/4.0/), which permits unrestricted use, distribution, and reproduction in any medium, provided the original work is properly cited.
(DOCX)
