# Supplementary material for: Effect of food swap recommendations on the healthfulness and carbon footprint of grocery purchases in a naturalistic online store: A randomized parallel trial
Source: PLoS Med. 2026 Aug 21;23(8):e1004847. doi: 10.1371/journal.pmed.1004847 (PMC13497253; doi:10.1371/journal.pmed.1004847)
Supplement: Supplementary file 1 [file pmed.1004847.s008.pdf]

Title : Online grocery store purchase
Approval Period: 04/10/2025 - 12/31/2999

Modification Form 2
Personnel Info 2
Participant Population 3
Study Location 3
General Checklist 4
Funding 5
Resources 5
Exempt Paragraph(s) 7
Purpose, Study Procedures 10
Participant Population(a-e) 16
Participant Population(f-i) 16
Risks 17
Privacy And Confidentiality 18
Conflict Of Interest 19
Consent Background 20
Assent Background 20
Attachments 21
Obligations 21

Title : Online grocery store purchase
Approval Period: 04/10/2025 - 12/31/2999

Modification

1. Summarize your proposed changes.

(a) We made minor edits to the consent form to update the expected date of the raffle and to indicate that we may post de-identified data to public repositories. This date has also be updated in the Protocol Information sections as appropriate.

(b) 0 participants have been enrolled to date.

(c) The study is closed to enrollment. We anticipate opening enrollment in late April, pending IRB approval.

(d) We have made minor edits to the consent form (see (a) above). However, no participants have been enrolled to date, so there is no need for re-consent as no participants have yet been contacted or consented.

(e) The revised consent form is attached in section 9.

2. Indicate Level of Risk

No Change

3. Update the Conflict of Interest (COI) section if any changes in COI have been made since the last protocol submission.

N Is there a change in the conflicting interest status for any existing personnel on this protocol?

Protocol Director

**Name**		**Degree (Program/year if student)**		**Position, e.g. Assistant Professor, Resident, etc.**
Anna H Grummon		PhD		Assistant Professor
**Department**		**Phone**		**E-mail**
Pediatrics - General Pediatrics		(650) 644-7366		agrummon@stanford.edu
**CITI Training current**				Y

Admin Contact

**Name**		**Degree (Program/year if student)**		**Position, e.g. Assistant Professor, Resident, etc.**
Amanda Zeitlin		MPH		Social Science Research Professional 1
**Department**		**Phone**		**E-mail**
Pediatrics - General Pediatrics		914-907-1447		abzeit@stanford.edu
**CITI Training current**				Y

Investigator

**Name**		**Degree (Program/year if student)**		**Position, e.g. Assistant Professor, Resident, etc.**
Cristina Lee		MPH		Social Science Research Professional
**Department**		**Phone**		**E-mail**
Pediatrics - General Pediatrics		(650) 644-7366		crisjlee@stanford.edu
**CITI Training current**				Y

Title : Online grocery store purchase
Approval Period: 04/10/2025 - 12/31/2999

Other Contact

**Name**<br>Kevin O'Sullivan		**Degree** (**Program/year if student**)<br>PhD (2025)		**Position, e.g. Assistant Professor, Resident, etc.**<br>Visiting Fellow
**Department**<br>Pediatrics		**Phone**<br>(650) 644-7366		**E-mail**<br>kevosull@stanford.edu
**CITI Training current**				Y

Academic Sponsor

**Name**<br>Emma Judith Walker		**Degree** (**Program/year if student**)<br>BA (2025)		**Position, e.g. Assistant Professor, Resident, etc.**<br>Research Assistant
**Department**<br>Pediatrics - General Pediatrics		**Phone**<br>(650) 644-7366		**E-mail**<br>awalker0@stanford.edu

Other Personnel

**Name**<br>Ashlyn Anderson		**Degree** (**Program/year if student**)<br>MPH		**Position, e.g. Assistant Professor, Resident, etc.**<br>Casual Employee
**Department**<br>Pediatrics - General Pediatrics		**Phone**<br>6506447366		**E-mail**<br>ashlynjanderson21@gmail.com
**CITI Training current**				Y

Participant Population(s) Checklist — Yes/No

- Children (under 18) N
- Pregnant Women and Fetuses N
- Neonates (0 - 28 days) N
- Abortuses N
- Prisoners N
- International Participants N
  Please enter the countries separated by comma
- Impaired Decision Making Capacity N
- Cancer Subjects N
- Laboratory Personnel N
- Healthy Volunteers N
- Students N
  Stanford students N Other students N
- Employees N
- Other (i.e., any population that is not specified above) Y

Study Location(s) Checklist — Yes/No

Title : Online grocery store purchase
Approval Period: 04/10/2025 - 12/31/2999

• Stanford University	Y
• Clinical & Translational Research Unit (CTRU)	N
• Stanford Medicine Health Care	N
Tri-Valley	N
• Stanford Medicine Children's Health	N
• VAPAHCS (Specify PI at VA)	
• Other (Specify other study locations)	

General Checklist

**Multi-site**	**Yes/No**
• Is this a multi-site study? A multi-site study uses the same protocol to conduct human subjects research at more than one site.	N

**Cooperative/Collaborative Study?**	**Yes/No**
• Are there any collaborating institution(s)? A collaborating institution is generally an institution that collaborates equally on a research endeavor with one or more institutions.	N

**Cancer Institute**	**Yes/No**
• Cancer-Related Studies (studies with cancer endpoints), Cancer Subjects (e.g., clinical trials, behavior/prevention) or Cancer Specimens (e.g., blood, tissue, cells, body fluids with a scientific hypothesis stated in the protocol).	N

**Tissues and Specimens**	**Yes/No**
• Human blood, cells, tissues, or body fluids (tissues)?	N
• Tissues to be stored for future research projects?	N
• Tissues to be sent out of this institution as part of a research agreement? For guidelines, please see Material Transfer Agreements	N
• Human Embryos or Gametes?	N

**Veterans Affairs** (**VA**)	**Yes/No**
• The research recruits participants at the Veterans Affairs Palo Alto Health Care System(VAPAHCS).	N
• The research involves the use of VAPAHCS non-public information to identify or contact human research participants or prospective subjects or to use such data for research purposes.	N

• The research is sponsored (i.e., funded) by VAPAHCS.	N
• The research is conducted by or under the direction of any employee or agent of VAPAHCS (full-time, part-time, intermittent, consultant, without compensation (WOC), on-station fee-basis, on-station contract, or on-station sharing agreement basis) in connection with her/his VAPAHCS responsibilities.	N
• The research is conducted using any property or facility of VAPAHCS.	N

**Payment**	**Yes/No**
• Subjects will be paid/reimbursed for participation? See payment considerations.	Y

**Funding**	**Yes/No**
• Training Grant?	N
• Program Project Grant?	N
• Federally Sponsored Project?	Y

Funding

Funding - Grants/Contracts/Agreements

**Funding Administered By :**	STANFORD	**SPO/RRA # (if available) :**	286556
**Grant # (if available) :**	5K01HL15860804	**Funded By/Partner (include pending) :**	National Institutes of Health
**Principal Investigator :**	Anna Grummon		

Grant/Contract Title if different from Protocol Title :
Developing and Evaluating Health and Environmental Messages to Improve Diet in Emerging Adults

For Federal projects, are contents of this protocol consistent with the Federal proposal?
Is this a Multiple Project Protocol (MPP)?
Is this protocol under a MPP?

Funding - Fellowships

Gift Funding

Dept. Funding

Other Funding

Resources :

a) Qualified staff.

Please state and justify the number and qualifications of your study staff.

Dr. Anna Grummon, PhD, MSPH, will serve as PI. She will oversee day-to-day operations of the project, including training and providing guidance to research assistants. Dr. Grummon will oversee survey development; she has extensive experience developing survey measures and using Qualtrics survey software to program surveys. Dr. Grummon will lead the analyses. Dr. Grummon holds a PhD and MSPH in Health Behavior from the University of North Carolina Gillings School of Global Public Health. She has previously designed survey instruments and analyzed the effects of messages and food labels on intentions, behaviors, and psychosocial outcomes; these experience have prepared her to lead this project.

Dr. Grummon's work will be supported by four additional lab members:

Amanda Zeitlin and Cristina Lee both have prior experience with similar research studies and are receiving detailed, project-specific training via in-person and Zoom meetings and via written materials. Ms. Zeitlin holds an MPH from USC and has previously worked on multiple NIH-funded studies at Stanford. Ms. Lee holds an MPH from Johns Hopkins and has experience with data analysis and public health research.

Kevin O'Sullivan is a PhD student at ETH Zurich and is a visiting researcher in Dr. Grummon's lab. He earned his bachelor's and master's degrees in Biomedical Engineering at Trinity College, University of Dublin. He has extensive expertise in programming and will build the study's online storefront under Dr. Grummon's guidance.

Ashe Walker is a fourth-year undergraduate student majoring in psychology at the University of West Virginia. They have previous experience working as a summer intern in Dr. Grummon's lab while working on an NIH-funded study.

In addition to the qualifications these RAs bring, study-specific training will be provided by Dr. Grummon. All RAs have also received a detailed written protocol explaining study procedures. Dr. Grummon has previously overseen research staff on similar food labeling studies at Stanford (IRB #67669 and #69580) and at her prior institutions (e.g., Harvard Longwood Campus IRBs #19-1790 and #21-0568) and is qualified to train staff in the tasks needed to carry out this project.

b) Training.

Describe the training you will provide to ensure that all persons assisting with the research are informed about the protocol and their research-related duties and functions.

Four research assistants (RAs) will assist with the project: Amanda Zeitlin, Cristina Lee, Ashe Walker and Kevin O'Sullivan. All RAs have prior experience with similar research and are receiving detailed, project-specific training via in-person and Zoom meetings and via written materials, provided by Dr. Grummon.

Training to support the study includes reviewing the project aim and goals, training on programming survey questions into Qualtrics, reviewing the proper storage of participant data, programming to build an online store, and effectively communicating with study vendors. Staff will also receive a detailed written protocol explaining study procedures. Dr. Grummon has previously overseen research staff on similar food labeling studies at Stanford (IRB #67669 and #69580) and at her prior institutions (e.g., Harvard Longwood Campus IRBs #19-1790 and #21-0568) and is qualified to train staff in the tasks needed to carry out this project.

c) Facilities.

Provide the location(s) **where the research will be conducted, including physical address if not conducted on site at Stanford University, Stanford Hospital on Pasteur Dr., Lucile Packard Children's Hospital on Welch Rd. or VAPAHCS. Describe the facilities and resources available to conduct the research at these sites.**

All study aspects will occur remotely. Participants will complete the online shopping task and survey questions at a location of their choosing.

d) Sufficient time.

Explain the time that you and your research team will allocate to perform the research activities, including data analysis.

We anticipate it will take ~12 months to achieve the two aims of the project including analyses. Dr. Grummon will devote at least 25% effort to this project. Ms. Zeitlin and Ms. Lee will each devote 10-20% effort to this project. Mr. O'Sullivan will devote 20% to the project. The timeframe and effort are consistent with our prior studies of similar nature.

e) Access to target population.

Explain and justify whether you will have access to a population that will allow recruitment of the required number of participants.

We will recruit a national convenience sample of 1,200 US adults, with an oversample of young adults ages 18-25 (to comprise 50% of total sample). Participants will be recruited by Prolific, an online participant recruitment platform with access to over 200,000 participants. Partnering with Prolific will allow us to easily reach the sample size we seek. Prolific carefully sources participants and uses vetting practices to prevent fraudulent respondents and ensure high data quality.

f) Access to resources if needed as a consequence of the research.

State whether you have medical or psychological resources available that participants might require as a consequence of the research when applicable. Please describe these resources.

NA, the need for psychological resources is not anticipated.

g) Lead Investigator or Coordinating Institution in Multi-site Study.

Please explain (i) **your role in coordinating the studies,** (ii) **procedures for routine communication with other sites,** (iii) **documentation of routine communications with other sites,** (iv) **planned management of communication of adverse outcomes, unexpected problems involving risk to participants or others, protocol modifications or interim findings.**

Title

Online grocery store purchase

Exempt Form

In order to qualify as Exempt, a protocol must be no more than minimal risk AND must only involve human subjects in one or more of the following paragraphs.

Select one or more of the following paragraphs:

1. N **Research, conducted in established or commonly accepted educational settings, that specifically involves normal educational practices that are not likely to adversely impact students opportunity to learn required educational content or the assessment of educators who provide instruction. This includes most research on regular and special education instructional strategies, and research on the effectiveness of or the**

comparison among instructional techniques, curricula, or classroom management methods.

2. N **Research that only includes interactions involving educational tests (cognitive, diagnostic, aptitude, achievement), adult surveys, and interviews, (includes group interviews/focus groups under 2ii only), or observation of public behavior (including visual or auditory recording) if at least one of the following criteria is met:**

- i) N The information obtained is recorded by the investigator in such a manner that the identity of the human subjects cannot readily be ascertained, directly or through identifiers linked to the subjects;
- ii) N Any disclosure of the human subjects responses outside the research would not reasonably place the subjects at risk of criminal or civil liability or be damaging to the subjects financial standing, employability, educational advancement, or reputation; or
- iii) N The information obtained is recorded by the investigator in such a manner that the identity of the human subjects can readily be ascertained, directly or through identifiers linked to the subjects, and an IRB conducts a limited IRB review to make the determination required by ? .111(a)(7)

3 Y **(i)Research involving benign behavioral interventions in conjunction with the collection of information from an adult subject through verbal or written responses (including data entry) or audiovisual recording if the subject prospectively agrees to the intervention and information collection and at least one of the following criteria is met: See (3)(ii) below for more on the definition of a benign behavioral intervention.**

- A) N The information obtained is recorded by the investigator in such a manner that the identity of the human subjects cannot readily be ascertained, directly or through identifiers linked to the subjects;
- B) Y Any disclosure of the human subjects responses outside the research would not reasonably place the subjects at risk of criminal or civil liability or be damaging to the subjects financial standing, employability, educational advancement, or reputation; or
- C) N The information obtained is recorded by the investigator in such a manner that the identity of the human subjects can readily be ascertained, directly or through identifiers linked to the subjects, and an IRB conducts a limited IRB review to make the determination required by ? .111(a)(7).

ii) **For the purpose of this provision, benign behavioral interventions are brief in duration, harmless, painless, not physically invasive, not likely to have a significant adverse lasting impact on the subjects, and the investigator has no reason to think the subjects will find the interventions offensive or embarrassing. Provided all such criteria are met, examples of such benign behavioral interventions would include having the subjects play an online game, having them solve puzzles under various noise conditions, or having them decide how to allocate a nominal amount of received cash between themselves and someone else.**

iii) **If the research involves deceiving the subjects regarding the nature or purposes of the research, this exemption is not applicable unless the subject authorizes the deception through a prospective agreement to participate in research in circumstances in which the subject is informed that he or she will be unaware of or misled regarding the nature or purposes of the research.**

Y **Is deception involved?**

4. N **Secondary research for which consent is not required: Secondary research uses of identifiable private information or identifiable biospecimens, if at least one of the following criteria is met:**

- i) N The identifiable private information or identifiable biospecimens are publicly available;
- ii) N Information, which may include information about biospecimens, is recorded by the investigator in such a manner that the identity of the human subjects cannot readily be ascertained directly or through identifiers linked to the subjects, the investigator does not contact the subjects, and the investigator will not re-identify subjects;
- iii) The research involves only information collection and analysis involving the investigator's use of identifiable health information when that use is regulated under 45 CFR parts 160 and 164, subparts A and E, for the purposes of "health care operations" or "research" as those terms are defined at 45 CFR 164.501 or for "public health activities and purposes" as described under 45 CFR 164.512(b); or
- iv) N The research is conducted by, or on behalf of, a Federal department or agency using government-generated or government-collected information obtained for nonresearch activities, if the research generates identifiable private information that is or will be maintained on information technology that is subject to and in compliance with section 208(b) of the E-Government Act of 2002, 44 U.S.C. 3501 note, if all of the identifiable private information collected, used, or generated as part of the activity will be maintained in systems of records subject to the Privacy Act of 1974, 5 U.S.C. 552a, and, if applicable, the information used in the research was collected subject to the Paperwork Reduction Act of 1995, 44 U.S.C. 3501 et seq.

5. N **Research and demonstration projects that are conducted or supported by a Federal department or agency, or otherwise subject to the approval of department or agency heads (or the approval of the heads of bureaus or other subordinate agencies that have been delegated authority to conduct the research and demonstration projects), and that are designed to study, evaluate, improve, or otherwise examine public benefit or service programs, including procedures for obtaining benefits or services under those programs, possible changes in or alternatives to those programs or procedures, or possible changes in methods or levels of payment for benefits or services under those programs. Such projects include, but are not limited to, internal studies by Federal employees, and studies under contracts or consulting arrangements, cooperative agreements, or grants. Exempt projects also include waivers of otherwise mandatory requirements using authorities such as sections 1115 and 1115A of the Social Security Act, as amended.**

- i) Each Federal department or agency conducting or supporting the research and (i) demonstration projects must establish, on a publicly accessible Federal Web site or in such other manner as the department or agency head may determine, a list of the research and demonstration projects that the Federal department or agency conducts or supports under this provision. The research or demonstration project must be published on this list prior to commencing the research involving human subjects.
- ii) [Reserved]

6. N **Taste and food quality evaluation and consumer acceptance studies:**

- i) N If wholesome foods without additives are consumed, or

ii) N If a food is consumed that contains a food ingredient at or below the level and for a use found to be safe, or agricultural chemical or environmental contaminant at or below the level found to be safe, by the Food and Drug Administration or approved by the Environmental Protection Agency or the Food Safety and Inspection Service of the U.S. Department of Agriculture.

7. Reserved for future use.

8. Reserved for future use.

1. Purpose

a) In layperson's language state the purpose of the study in 3-5 sentences.

Aim 1: Experts agree that the US diet must change substantially to reduce the burden of diet-related diseases and mitigate environmental degradation. One way to support this change is to encourage consumers to make simple, achievable swaps in which they replace one food with another similar food that is healthier and less damaging to the environment. This study aims to determine whether receiving recommendations for healthier or more environmentally sustainable food swaps (or both) in an online grocery store environment improves the healthfulness and environmental sustainability of consumers' food and beverage purchases compared to shopping as usual without swap recommendations.

Aim 2: Researchers and advocates have argued that adopting nutrition standards for carceral facilities could improve population health; however, it is unclear whether the U.S. public would support such a policy. Understanding how to increase public support for carceral food policies, including demographic predictors of support, could inform campaigns and other messaging to generate support for these policies. This research aims to determine if the rationale given for adopting nutrition standards in carceral facilities affects support for this policy and what demographic characteristics are predictors of policy support.

b) State what the Investigator(s) hope to learn from the study. Include an assessment of the importance of this new knowledge.

Aim 1: Poor diet quality accounts for 500,000 deaths and more than $50 billion in healthcare costs in the US every year, and food production accounts for up to one-third of all human-caused greenhouse gas emissions and is a major driver of freshwater depletion, land use, and biodiversity loss. Broad recommendations from dietary guidelines, academic reports, and nonprofits can be challenging for consumers to implement day-to-day, especially given the complexity of food choice.

Encouraging simple dietary swaps has the potential to improve diet quality and reduce the environmental harms from food production because otherwise similar foods – e.g., a beef burger and a turkey burger – can have substantially different nutritional profiles and environmental impacts. Moreover, swaps may be easier and more attractive for consumers to implement than more extensive dietary changes, such as eliminating meat or animal products. Swap recommendation systems have become more popular, and questions remain about their impacts. First, although studies suggest that recommending healthier food swaps can reduce how much sodium, saturated fat, or calories consumers purchase, it remains largely unknown whether recommending more environmentally sustainable swaps (e.g., recommending foods with lower carbon footprints) will lead consumers to purchase more environmentally sustainable foods. Second, most swap recommendation systems focus on only one dimension of a food's impacts (i.e., just healthfulness or just environmental sustainability), and it is unclear how recommending swaps meant to improve one dimension will affect outcomes on the other dimension. Health-focused swaps may have positive spillovers on environmental sustainability and vice versa (i.e., a win-win), given the overlap between healthy and environmentally sustainable dietary patterns. On the other hand, it is also possible that health-focused swaps could negatively impact environmental sustainability and vice versa (i.e., a win-lose) because some

healthier foods are relatively unsustainable (e.g., low-fat dairy products) and some sustainable foods are relatively unhealthy (e.g., some meat mimic products). Third, given the potential tradeoffs between health and environmental sustainability, swap recommendation systems might wish to address both dimensions simultaneously, for example, by only recommending healthier swaps that meet a minimum environmental sustainability threshold and vice versa. However, the effects of such a system remain unknown.

To address these gaps, our randomized controlled trial will determine whether providing recommendations for healthier or more environmentally sustainable swaps (or both) in an online grocery store environment improves the healthfulness and environmental sustainability of food and beverage purchases.

Aim 2: Nearly 2 million individuals in the U.S. are incarcerated, and these individuals are at increased risk for poor health outcomes, including high blood pressure and cancer, compared to the general U.S. population. The health of the incarcerated population is also an equity concern, given historically marginalized groups, including Black, Indigenous, and Latino/a/x individuals are incarcerated at disproportionate levels in the U.S.

Research has demonstrated that access to adequate nutrition inside of carceral facilities is limited, and that meals served are often insufficient to meet incarcerated individuals' caloric and nutrient needs. In particular, few, if any, carceral facilities require meals to adhere to nutrition standards, such as the U.S. Department of Agriculture Dietary Guidelines for Americans, despite this being mandatory in other U.S. institutions, including schools.

While researchers and advocates support the adoption of nutrition standards for carceral facilities, it is important to determine if the U.S. public would support such a policy. Understanding how to increase public support for carceral food policies could inform campaigns and other messaging to generate support for these policies. Additionally, understanding demographic predictors of support for such a policy could help identify natural groups of champions of the policy (and likewise identify groups that may oppose such a policy).

c) **Explain why human subjects must be used for this project.** (**i.e. purpose of study is to test efficacy of investigational device in individuals with specific condition; purpose of study is to examine specific behavioral traits in humans in classroom or other environment**)

The purpose of this study is to (1) evaluate adults' food choices in response to receiving recommendations for healthier or more environmentally sustainable swaps, and (2) determine the views of adults on support for a new policy introducing nutrition standards for carceral facilities. To achieve these goals, we need to examine specific responses of humans to these recommendations and policies. Therefore, human subjects must be used for this project.

2. Study Procedures

a) **i**) **Describe ALL the procedures human participants will undergo. ii**)**Are the research procedures the least risky that can be performed consistent with sound research design? iii**)**For research involving collaborators, please specify the respective roles of Stanford and each collaborator on the protocol.**

i)
Recruitment:
We will recruit participants through Prolific, a survey research firm. Prolific contains a proprietary panel of over 200,000 highly vetted participants. Prolific runs onboarding checks based on attention, comprehension, and honesty to ensure high-quality data and vet the weakest responses. In addition, Prolific checks IP addresses and then verifies identities with Onfido's bank-grade ID checks. Our research team will use Prolific's filtering system to ensure only eligible panelists are contacted. We will create a message that will be sent to eligible Prolific panelists (i.e., 18 years or older, living in the US), including a study title, brief study description, and link to the survey (see attached message in section 11). Prolific will notify eligible panelists about the study through their chosen method (e.g., email, posting on panelists' personal Prolific

study page). Participants can read the study description and choose to opt-in to take the study.

Screening:
Participants must be age 18 or older and reside in the US to participate. Participants will have been screened through a filtering process on Prolific's site. In addition, before entering the survey, they will take a two-question screener asking their age and country of residency. Participants who are ineligible based on their responses to these questions will be re-directed to the survey termination page.

Consent:
For eligible participants, the first page of the first survey will display this study's electronic consent form. The consent form will inform potential participants that deception may be used in studies and that agreeing to participate in this study includes agreeing to possible deception. The consent form will also explain that if deception is used participants will be debriefed at the end of the study. If participants are interested in participating, they will indicate their consent by clicking "I consent" on the form.

Experiment:
Participants will complete three ~15-minute remote study visits, each one week apart.

VISIT 1
In the first study visit, participants will provide electronic informed consent as described above. Then, participants will engage in an online shopping task. The participants will enter a naturalistic online grocery store developed for this study. The online grocery store provides an immersive experience that mimics a real online grocer's look, feel, and functionality. The online supermarket was developed using the MERN stack and is hosted on servers located in the U.S. The store will include products across 4 core departments (Beverages, Grocery, Frozen, and Meat). Participants will navigate the store by clicking on the departments and categories or by using a search bar, similar to a real online store. Participants will be able click on items to view additional details (e.g., the Nutrition Facts Label).

Participants will be instructed to shop as they usually would for items in the following food groups: beverages, boxed and frozen meals, milk and dairy products, proteins, soups, and sweets and snacks. We chose to focus on these categories because they are top contributors to saturated fat, added sugar, sodium, calorie intake, or the overall dietary carbon footprint in the US. These categories also offer variation in both healthfulness and carbon footprint across products.

All participants will shop in a "pretend" online grocery store. Participants will be given a budget of $50 to shop using "pretend" money. To check out of the store, participants must spend between 0.5 and 1.5 times this budget but otherwise will be free to shop as they wish. To motivate participants to behave as they would in an actual store, we will inform them that all participants will be entered into a raffle and 25 (i.e., 1 in 50) will be randomly chosen to have their selections from 1 week of the study delivered to them, plus the remainder of the shopping budget from that week. At the end of study visit 3, we will reveal that, instead of receiving groceries, these randomly selected participants will receive $50 (the budget for the shopping task) to their PayPal account distributed by Prolific.

After completing the survey questions for Aim 1, participants will complete the survey questions for Aim 2. First, all participants will read the following prompt: "The US government has defined what meals are considered healthy. Imagine that a new law required US prisons to serve only meals that meet this definition." One in four participants will read the prompt with no additional information. A second group (also to comprise 1/4 of the sample) will read the prompt plus the following statement, "The goal of this new law is to make communities safer. Prisoners who return home healthy are more likely to be employed and less likely to commit crimes." A third group (1/4 of the sample) will read the prompt plus the following statement, "The goal of this new law is to save the government money. Serving healthier meals to prisoners would improve their health, which would lower healthcare costs." Finally, a fourth group will read the prompt plus the following statement, "The goal of this new law is to ensure the right of all people to have access to healthy food, whether or not they are in prison." The survey software will randomly assign participants across these statements. Then, all participants will be asked about their support for this policy

on a scale of 1, "Strongly oppose this law" to 5, "Strongly support this law."

No activities currently or in the future under this protocol will target inclusion of incarcerated individuals.

Finally, after participants have completed the survey questions for Aim 1 and Aim 2, they will be asked about their demographic questions (e.g., gender, education, income), experience with criminal justice, and health and environmental motivation.

Prolific will create participant IDs for all participants who complete the first study visit. Our study team will export and download the IDs from Prolific's site.

VISIT 2
Approximately one week later, our research team will use Prolific to contact the same participants to complete the second study visit. On Prolific's site, we will enter the participant IDs so only those participants who have completed in the first study visit will be contacted with a link to complete the follow-up study visit. Using an ID generated by Prolific allows our team to access the same participants without receiving any identifying information.

In Visit 2, participants will complete the same shopping task and surveys as for Aim 1 above. (They will not repeat Aim 2 questions or demographics - those will only be measured at Visit 1).

For the shopping task, participants will receive the same instructions as those described during the first study visit. Then, participants will be randomized to 1 of 4 trial arms: 1) Health Swaps, 2) Environment Swaps, 3) Both Health and Environment Swaps, or 4) Control.

In the Health Swaps arm, the online grocery store will display “health grade” labels on all products indicating their healthfulness as estimated by UK Ofcom Nutrient Profiling Model scores. The health score labels will mimic Nutri-Score labels, a labeling system used in some European countries, showing a color-coded grade of “A” (green) through “F” (red) on each product. We will apply "A" and "B" labels to products that meet the UK's cutoff for products that can be marketed to children and "C", "D" and "F" labels to products less healthy than this cutoff (based on tertiles of Ofcom scores within each food group). When participants attempt to add a less healthy product to their cart (e.g., "D" or "F" health label), the store will automatically suggest a healthier product from the same category (e.g., with a "A" or "B" health label).

In the Environment Swaps arm, the online grocery store will display “climate grade" labels on all products indicating their climate impact. We will calculate climate impact as the greenhouse gas emissions associated with producing the product in carbon dioxide equivalents (CO_2-eq) per 100g (i.e., “carbon footprint”). We will apply label based on quintiles of carbon footprints in each food group. When participants attempt to add a high-climate-impact product to their cart (e.g., with a "D" or "F" climate label), the store will automatically offer them swaps to more environmentally sustainable products (e.g., with a "A" or "B" climate label).

In the Health and Environment Swaps arm, the online grocery store will display both the health and climate grade labels on all products. When participants attempt to select add a product with a “D” or “F” label on either dimension to their cart, the store will automatically offer them swaps to products that offer improvement over the original food on at least 1 dimension (health or sustainability) and were at least as good or better on the other dimension, with the additional guardrail that the store never suggests products with a “D” or “F” label on either dimension.

In the Control arm, the online grocery store will not display any extra labels or offer any swaps.

As in the first study visits, after completing the shopping task, participants will answer questions about their shopping experience (e.g., "While you were shopping, did you notice any nutrition-related labels?").

VISIT 3

Approximately one week later, our research team will use Prolific to contact the same participants to complete the third study visit. All participants who completed visit 1 will be eligible, even those who did not complete visit 2. On Prolific's site, we will enter the participant IDs so only those participants who have completed in the first study visit will be contacted with a link to complete the follow-up study visit. Using an ID generated by Prolific allows our team to access the same participants without receiving any identifying information.

Participants will receive the same shopping task instructions as those described during the first and second study visit. They will remain in the trial arm they were randomized to in Visit 2. They will complete the shopping task and online survey as in the prior visits.

At the end of the study, (as described above) we will debrief the participants about the study purpose and reveal that instead of receiving groceries, participants who are selected from the raffle will receive $50 to their PayPal account distributed by Prolific (see Debrief attached in section 11).

There will be no follow-up after the third study visit is complete.

ii) Yes, the research procedures are the least risky that can be performed consistent with sound research design (see response 2b).

iii) NA; we will not be collaborating on this project.

b) Explain how the above research procedures are the least risky that can be performed consistent with sound research design.

The goal of this study is to (1) determine whether providing recommendations for healthier or more environmentally sustainable swaps (or both) in an online grocery store environment improves the healthfulness and environmental sustainability of food and beverage purchases (2) determine policy support for a law requiring nutrition standards in carceral facilities. Online surveys represent the least risky procedure for addressing this objective: the main risks to participants are (a) boredom while taking the survey, (b) minor psychological distress that may result from considering adverse effects of choosing unhealthy foods (c) minor psychological distress that may result from considering the adverse effects of choosing foods that contribute to carbon emissions (d) minor psychological distress from thinking about carceral facilities; and (e) accidental disclosure of data collected during the survey. We will attempt to reduce the risk of any psychological harm by keeping the survey short (limited to approximately 15 minutes) and asking only questions necessary to address the research objectives. We will reduce the risk of accidental disclosure by only asking non-sensitive questions such that if participants' responses were accidentally disclosed, this would not pose harm to their reputation, employability, insurability, etc. Moreover, the study team will keep raw participant data in a secure, password protected server. Finally, we are not collecting and will not have access to any identifying information about participants. Therefore, if data are accidentally disclosed, it will not be possible to link responses to participants. We therefore believe these procedures are the least risky that can be performed consistent with a sound research design.

c) State if deception will be used. If so, provide the rationale and describe debriefing procedures. Since you will not be fully informing the participant in your consent process and form, complete an alteration of consent (in section 9). Submit a debriefing script (in section 11).

For Aim 1, to incentivize participants to behave as they would in a real store, we will inform them that 25 would be randomly chosen to have their selections delivered to them, plus the remainder of the shopping budget. At the end of the study, we will debrief participants on the study purpose and inform them that these participants will instead receive a $50 to their PayPal account distributed by Prolific. Using this very minor deception allows us to incentivize participants to behave as they normally would when choosing products from an online grocery store to minimize potential social desirability bias introduced by participating in a study while avoiding having to collect their addresses to ship items. Moreover, we expect that most participants will prefer to receive money over their selected products, as the money can be used to purchase any items, including beverages or snacks, and is the customary method for payment to Prolific panelists.

Title : Online grocery store purchase
Approval Period: 04/10/2025 - 12/31/2999

After completing the survey, participants will receive a debriefing form explaining this minor deception (see attached Debriefing Materials).

d) State if photo, audio or video recording will occur. Describe what will become of the photos or recording after use, e.g., shown at scientific meetings, erased. Describe the final disposition of the recordings.

N/A

3. Background

a) Describe past findings leading to the formulation of the study.

Aim 1:
Many consumers report wanting to eat healthier, more environmentally sustainable diets. However, consumers struggle to realize these goals, perhaps partly because many find it hard to understand what foods are healthier and more environmentally sustainable. Dietary guidelines, academic reports, and nonprofits offer broad recommendations about what to eat – for example, recommending diets high in fruits, vegetables, and legumes and low in red and processed meats. However, these broad recommendations can be challenging for consumers to implement day-to-day, especially given the complexity of food choice.

One way to address this challenge is to encourage consumers to make simple, achievable swaps in which they replace one food with another similar food. Encouraging swaps has the potential to improve diet quality and reduce the environmental harms from food production because otherwise similar foods – e.g., a beef burger and a turkey burger – can have substantially different nutritional profiles and environmental impacts. Moreover, swaps may be easier and more attractive for consumers to implement than more extensive dietary changes, such as eliminating meat or animal products. Swaps could also increase self-efficacy (a key antecedent to long-term behavior change) because they involve making small, graded changes.

Recently, it has become possible to recommend swaps to consumers in real-time when they decide what foods to buy. The US grocer Kroger, for example, offers a program called "OptUp," which recommends swaps from less healthy to similar but more healthy foods. Similarly, apps like "LiveWell" allow consumers to scan foods' barcodes with their phones to receive recommendations for swaps to similar but healthier or more environmentally sustainable foods. Industry reports indicate that consumers are increasingly interested in using these swap recommendation systems.

Even as swap recommendation systems become more popular, questions remain about their impacts. We, therefore, aim to determine the effectiveness of providing recommendations for healthier or more environmentally sustainable swaps (or both) improves the healthfulness and environmental sustainability of food and beverage choices.

Aim 2:
Research has demonstrated that access to adequate nutrition inside carceral facilities is limited and that meals served are often insufficient to meet incarcerated individuals' caloric and nutrient needs. In particular, few, if any, carceral facilities require meals to adhere to nutrition standards, such as the U.S. Department of Agriculture Dietary Guidelines for Americans, despite this being mandatory in other U.S. institutions, including schools.

Research and advocates have expressed support for a policy requiring carceral facilities to adhere to U.S. government dietary guidelines. However, it is unclear whether the U.S. public would support such a policy. Research has demonstrated that the rationale given for a policy can influence public support. In particular, moral, economic, and public safety arguments have been shown to affect public policy support. Our study aims to build upon the existing literature to examine how the rationale given for a policy to adopt nutrition standards in prisons affects public support for this policy. We will examine three rationales: public safety,

cost, and the human right to healthy food.

4. Participant Population

a) State the following: (i) the number of participants expected to be enrolled at Stanford-affiliated site(s); (ii)the total number of participants expected to enroll at all sites; (iii) the type of participants (i.e. students, patients with certain cancer, patients with certain cardiac condition) and the reasons for using such participants.

(i) We will enroll ~1200 participants for an online study. (ii) NA, no participants will be enrolled elsewhere. (iii) Participants will be healthy adults ages 18 and older. We will use quota sampling to ensure that approximately 50% of participants are emerging adults ages 18-25. We are recruiting adults because they are primarily responsible for grocery store purchases. We are oversampling for emerging adults because we are interested in assessing whether effects differ by age group.

b) State the age range, gender, and ethnic background of the participant population being recruited.

We will recruit adults ages 18+ who reside in the US. We will use quota sampling to ensure that approximately 50% of participants are emerging adults ages 18-25. We anticipate the race, gender, and ethnic background will approximately match that of the US overall.

c) State the number and rationale for involvement of potentially vulnerable subjects in the study (including children, pregnant women, economically and educationally disadvantaged, decisionally impaired, homeless people, employees and students). Specify the measures being taken to minimize the risks and the chance of harm to the potentially vulnerable subjects and the additional safeguards that have been included in the protocol to protect their rights and welfare.

N/A, we will not be recruiting potentially vulnerable subjects.

d) Describe your plan to identify and recruit potential participants including who will inform them about the study and how they will be initially contacted by the researchers (e.g., Participant Engagement services; chart review; treating physician; ads including social media posts). All final or revised recruitment materials must be approved by the IRB before use. Contacting potential participants is not permitted prior to IRB approval. See Recruitment Guidance for additional information.

We will recruit participants through Prolific, a survey research firm. Prolific has a proprietary pool of over 200,000 vetted participants. Our study team will use Prolific's filtering system to select participants 18 + who reside in US. Prolific will recruit participants by notifying eligible panelists of the study opportunity through their preferred communication method (e.g., messages on their Prolific study page, email, or instant notification through Chrome extension). Eligible panelists will choose to opt-in to take the survey by clicking on the Qualtrics hyperlink in the invitation provided by our study team. The link will take them to a Qualtrics survey containing this study's tasks and survey items. To avoid sample selection bias based on the survey topic, our team has created a general message (see message in section 11), including a short study title and brief description, the study length and reward amount and type, and the opt-in link.

e) Inclusion and Exclusion Criteria.

Identify inclusion criteria.

Participants must be aged 18 or older to participate. They must speak English, reside in the US, and have access to the internet to complete the online survey.

Identify exclusion criteria.

We will exclude participants who are outside the target age range (minors), who do not speak English, who do not reside in the US, and who do not have computer and Internet access. This study is excluding non-English speakers because the surveys will be available in English only, and non-English speakers would have difficulty answering the survey questions.

f) Describe your screening procedures, including how qualifying laboratory values will be obtained. If you are collecting personal health information prior to enrollment (e.g., telephone screening), please

request a limited waiver of authorization in section #10.

We will use Prolific's filtering system to request only panelists residing in the US and over age 18 to be contacted. In addition, we will set up the Qualtrics survey to only allow participants with US-based IP addresses to access the survey. Before consenting, the participants will be asked two screening questions about their age and country of residence. Those younger than 18 and/or residing outside the US will be automatically re-directed to the survey's termination page. There are no other screening procedures. No PHI is being collected.

g) **Payment/reimbursement. Explain the amount and schedule of payment or reimbursement, if any, that will be paid for participation in the study. Substantiate that proposed payments are reasonable and commensurate with the expected contributions of participants and that they do not constitute undue pressure on participants to volunteer for the research study. Include provisions for prorating payment. See payment considerations**

Prolific will manage all incentives and compensation. Participants are compensated through monetary awards to their PayPal accounts in US dollars. Participants will receive $4.50 for the first study visit, $6.00 for the second visit, and $8.00 for the third visit. The increasing incentive amount for later study visits reflects participants' increasing involvement in the study.

In addition, we will conduct a raffle on or around June 14th in which selected participants will receive an additional $50 sent by Prolific to their PayPal account within one month of the study close. Participants will have a 1 in 50 chance of receiving this payment (i.e., 25 participants will be selected). The consent form contains information describing the raffle, but instead of explaining the prize of $50, we state that participants who are randomly selected to win the raffle will receive up to $50 worth of groceries. The participants will be debriefed at the end of the study (i.e., after completing study visit 3) that the raffle winners will receive $50 distributed to their PayPal account rather than groceries.

These incentive amounts were determined by the research team based on feedback from Prolific about incentives for similar studies and will be agreed upon between the participant and Prolific prior to the participant beginning the survey.

h) **Costs. Please explain any costs that will be charged to the participant.**

NA, no costs will be charged.

i) **Estimate the probable duration of the entire study. Also estimate the total time per participant for: (i) screening of participant; (ii) active participation in study; (iii) analysis of participant data.**

The probable duration of the entire study is 12 months. For participants, total time is ~46 minutes: (i) screening will take <1 minute, (ii) active participation in the 3 study visits will take a total of 45 minutes, including ~10 minutes to complete the online shopping tasks and 5 minutes to complete the survey questions x 3 study visits. (iii) Analysis of participant data will take the investigators ~6 months (this time is not counted toward participant time-in-study because participants will not be re-contacted after they complete the second survey). We then anticipate writing manuscripts related to the research findings over the remaining duration of the study.

5. Risks

a) Describe risks. Include risks to privacy, confidentiality, etc..

The main privacy risk to participants is accidental disclosure of data collected during the survey. Not only is the likelihood of accidental disclosure low due to the steps we will take to reduce these risks (see Question 2b), but the risks of such disclosure to participants are minimal, as the data will pertain to their reactions to suggestions for product replacements during an online grocery store purchasing task and their reactions to that shopping experience (e.g., which items they choose to purchase and how they felt about their choices) as well as their opinion on policy support for nutrition standards.

b) **If you are conducting international research, describe the qualifications/preparations that enable you**

to both estimate and minimize risks to participants. Provide an explanation as to why the research must be completed at this location and complete the International Research Form. If not applicable, enter N/A.

N/A

c) Could any disclosure of the participant's response outside the research reasonably place them at risk of loss of insurability, criminal or civil liability, or be damaging to the participant's financial standing, employability, or reputation?

No. The data will pertain to participants' reactions and choices regarding online grocery store purchasing as well as views about nutritional standards in and relationship to carceral facilities; their responses are therefore unlikely to place them at risk of loss of insurability, criminal or civil liability, or be damaging to the participant's financial standing, employability, or reputation.

6. Benefits

a) Describe the potential benefit(s) to be gained by the participants or by the acquisition of important knowledge which may benefit future participants, etc.

Participants may increase their knowledge of the healthfulness and environmental sustainability of various food items and which similar foods may be healthier and more sustainable. Participants may also experience personal satisfaction from knowing they have contributed to a research project to evaluate the impact of health and environmental labels to improve the population's dietary quality and reduce carbon emissions. Potential benefits to society include developing effective health and/or environmental labels for informing consumers and information on whether including suggestions for alternative products results in healthier and more sustainable food purchases. Participants may also feel satisfaction from knowing they have contributed to a research project to evaluate policy support for federal nutrition standards in carceral facilities. This may potentially benefit society by leading to effective strategies to garner support among the US population for laws that would allow those in carceral facilities to eat a healthier diet.

7. Privacy and Confidentiality

Privacy Protections

a) Describe the setting and method (e.g. crowded waiting room, patient exam room, telephone or email communication) in which interactions will occur and how the privacy interests of participants will be maintained. Note, high risk data such as PHI must be sent via "Secure:" email per Stanford policy.

Prolific will recruit participants by sending notifications to participants based on participants' preferred method of contact (e.g., a Prolific desktop extension, email) and by posting the study on their personalized Prolific Study Page. Participants can read the study title and study description (see section 11, Attachments) and choose to opt in by clicking on the Qualtrics study link. Aside from creating a study description, the study team will not be involved in any other aspect of recruitment and will not have access to participants' contact information.

Participants will complete the survey online from a location of their choosing.

Prolific will provide participants with incentives for completing the survey. The study team will not be involved in disbursing incentives.

Confidentiality Protections

b) Specify the PHI (protected health information) or other individually identifiable data or specimens you will obtain, use or disclose to others. PHI is health information linked to one or more of the HIPAA identifiers listed above. List BOTH health information AND identifiers.

No health information or other individually identifiable data will be collected (i.e., there will be no PHI involved in this study). As can be seen in the attached survey measures document, none of the survey questions involve PHI. Qualtrics will be configured to not collect participant IP addresses. Additionally, Prolific will be responsible for recruitment and will only reveal anonymous participant IDs to our study team. We will use these participant IDs to request Prolific notify the same participants to complete the second and third study visits. We will not have access to the key linking the IDs to the participants.

c) **Describe:** (**i**) **how data will be maintained** (**e.g., paper or electronic spreadsheet, desktop computer, laptop or other portable device**); (**ii**) **how you will maintain the confidentiality and data security,** (**e.g., password protected computer, encrypted files, locked cabinet and office**); **and** (**iii**) **who will have access to the data** (**e.g., research team, sponsors, consultants**)

(i) Qualtrics will automatically capture participants' survey responses. The study team will be able to download these responses from Qualtrics. As stated in 6b, responses are anonymized because we are not collecting any identifiable data. We will store participant responses in an electronic spreadsheet (i.e., Excel file) in a password-protected shared server. (ii) To maintain confidentiality and data security, we will only allow members of the study team to have access to the server that contains the data, and the server will be password-protected. As described above, we have also structured the survey so that the data do not include any identifying information. (iii) Only members of the study team will have access to the raw data. Although we expect the raw data will already be anonymized based on the nature of the data collection activities, the study team will review the raw data for disclosure risk and remove any unexpected identifiers (e.g., from participants' responses to free response questions). We will use secure servers (e.g., Box or OneDrive) to share anonymized data with co-investigators during analysis. After analysis, we will upload the anonymized data to a public repository.

d) **If sharing data with others, describe how data will be transferred or transmitted** (**e.g., file transfer software, file sharing, email**). **If transmitted via electronic networks, confirm a Stanford University IT approved platform will be used** (**see https://uit.stanford.edu/guide/riskclassifications**) **or that data will be encrypted while in transit. Additionally, confirm appropriate agreements are in place to allow for the sharing** (**see https://ico.stanford.edu/stanford-researchers/who-will-handle-my-agreement**). **If using or sharing PHI, refer to the following policies: https://uit.stanford.edu/security/hipaa.**

N/A

e) **If you plan to code the data, describe the method in which it will be coded and indicate who will have access to the key to the code.**

N/A

f) **How will you educate research staff to ensure they take appropriate measures to protect the privacy of participants and the confidentiality of data or specimens collected** (**e.g. conscious of oral and written communications, conducting insurance billing, and maintaining paper and electronic data**)**?**

Research staff will be required to complete CITI training. The PI will additionally provide in-person and online training on privacy and confidentiality, including walking through scenarios with RAs regarding proper ways to communicate orally and in writing, proper ways to maintain paper and electronic data, and computer security.

8. Potential Conflict of Interest

Investigators are required to disclose any outside interests that reasonably appear to be related/li to this protocol.

Outside Interest Tasks

Investigators	Role	Potential COI?	Date Outside Interest Answered	Date OPACS Disclosure Submitted	COI Review Determination
Anna H Grummon	PD	N	08/15/2024		N/A

9. Consent Background

If the study involves an interaction with participants, attach an information sheet with your study details by clicking the ADD button below.

9. 1 Consent Information Sheet

Sponsor's Consent Version Number: (**if any**) **:**

a) Describe the informed consent process. Include the following.

- **i) Who is obtaining consent?** (**The person obtaining consent must be knowledgeable about the study.**)
- **ii) When and where will consent be obtained?**
- **iii) How much time will be devoted to consent discussion?**
- **iv) Will these periods provide sufficient opportunity for the participant to consider whether or not to participate and sign the written consent?**
- **v) What steps are you taking to minimize the possibility of coercion and undue influence?**
- **vi) If consent relates to children and if you have a reason for only one parent signing, provide that rationale for IRB consideration.**

(i) This is an online study and consent will be taken online. (ii)The first page of the survey will include the Information Sheet (attached), and participants will only be allowed to proceed to the study if they choose to participate by selecting "I consent." (iii) Participants can take as much time as they wish to read the consent information and decide if they wish to participate. (iv) Participants will be recruited from a Prolific's opt-in panel. Those who read the recruitment message and choose to proceed will receive the survey. All incentives are commensurate with amounts used in similar studies.

b) What is the Procedure to assess understanding of the information contained in the consent? How will the information be provided to participants if they do not understand English or if they have a hearing impairment? See HRPP Chapter12.2 for guidance.

Participants have been vetted by Prolific before recruitment. Only participants who understand and can read English proficiently will be recruited. In addition, our Information Sheet (i.e., consent form) is written in plain language to be easily understood.

c) What steps are you taking to determine that potential participants have the capacity to participate in the decision-making process? If your study may enroll adults who are unable to consent, describe (**i**) **how you will assess the capacity to consent,** (**ii**) **what provisions will be taken if the participant regains the capacity to consent,**(**iii**) **who will be used as a legally authorized representative, and** (**iv**) **what provisions will be made for the assent of the participant.**

Participants have been vetted by Prolific before recruitment. We are only recruiting adults who are able to consent.

10. Assent Background (less than 18 years of age)

11. Attachments

Attachment Name	Attached Date	Attached By	Submitted Date
Graphics used in study	10/04/2024	abzeit	
Debriefing statement	11/09/2024	abzeit	
Recruitment message (updated)_Revised	01/06/2025	abzeit	
Swaps Survey Baseline_Revised	01/06/2025	abzeit	
Swaps Study Visit 2 Revised	01/06/2025	abzeit	
Swaps Study Visit 3 Revised	01/06/2025	abzeit	

Obligations

The Protocol Director agrees to:

- Adhere to principles of sound scientific research designed to yield valid results
- Conduct the study according to the protocol approved by the IRB
- Be appropriately qualified to conduct the research and be trained in Human Research protection, ethical principles, regulations, policies and procedures
- Ensure all Stanford research personnel are adequately trained and supervised
- Ensure that the rights and welfare of participants are protected including privacy and confidentiality of data
- Ensure that, when de-identified materials are obtained for research purposes, no attempt will be made to re-identify them.
- Disclose to the appropriate entities any potential conflict of interest
- Apply relevant professional standards.
- Any change or modification in the research protocol must be submitted to and approved by the IRB prior to the implementation of such change, except when necessary to eliminate apparent immediate hazards to the participant.
- For studies with expiration dates, submit a Continuing Review prior to the end of the approval period. An IRB Continuing Review (Renewal) Notice to Renew Protocol is sent to the Protocol Director prior to the expiration date of the protocol.
- Report promptly any new information, complaints, possibly serious and/or continuing noncompliance, or unanticipated problems involving risks to participants or others.

All data, including signed consent forms when applicable, must be retained for a minimum of three years past the completion of the research. Additional requirements may be imposed by your funding agency, your department, or other entities (e.g. 6 years for studies conducted under HIPAA or VAPAHCS). (See also Research Policy Handbook Retention of and Access to Research Data)

Questionnaries and Interview Guide

Y **By checking this box, I verify that I, as the Protocol Director (PD) responsible for this research**

protocol, have read and agree to abide by the above obligations, or that I have been delegated authority by the PD to certify that the PD has read and agrees to abide by the above obligations.

APPROVAL LETTER/NOTICE NOTE: List all items (verbatim) that you want to be included in your approval letter (e.g., Amendment date, Investigator's Brochure version, consent form(s) version(s), advertisement name, etc.) in the box below.

Title: Food Swaps to Improve the Healthfulness and Reduce the Carbon Footprint of Grocery Purchases
Protocol Date: April 10, 2025
NCT#: NCT06648226

Study Principal Investigator:

Anna H. Grummon
Stanford University
email: agrummon@stanford.edu

Introduction

The goal of the analyses described here is to use data we collected through an online randomized trial to examine consumer responses to receiving health, climate, or health and climate swap recommendations while grocery shopping. This analysis plan pre-specifies the analyses before collecting data and therefore serves as our ex-ante planned analysis.

Study Protocol

Participants will complete an online randomized trial. Participants will complete 3 online study visits, each separated by ~1 week. For all 3 study visits, participants will complete a shopping task in a naturalistic online grocery store and respond to survey questions. In the first study visit (baseline), participants will provide electronic informed consent and then complete the shopping task with no modifications to the store. At the end of the first visit, participants will be randomized to 1 of 4 trial arms: 1) health swaps, 2) climate swaps, 3) combined health and climate swaps, or 4) control. For the second and third study visit, participants will complete the shopping task in their randomly assigned trial arm, then respond to survey questions.

In the shopping task, participants will be instructed to shop as they usually would for items in the following food groups: beverages, boxed and frozen meals, dairy and dairy alternatives, meat and meat alternatives, soups, and sweets and snacks. They will be given a budget of $50. To checkout, participants need to have spent between 0.5 and 1.5 times this budget (i.e., $25 to $75), but otherwise will be free to shop as they wish. To incentivize participants to behave as they would in a real store, we will inform them that 25 will be randomly chosen to have their selections delivered to them, plus the remainder of the shopping budget as an electronic gift card. At the end of the study, we will debrief participants that those who were selected for delivery would instead receive an electronic gift card for $50.

Statistical Considerations

General Principles

We will use a two-sided critical alpha of 0.05 to conduct all statistical tests. All confidence intervals presented will be 95% and two-sided. Unless otherwise noted, analyses will be intent-to-treat, examining all participants in their randomized groups (regardless of the extent of the study they complete after randomization).

Co-Primary Outcomes

There are two co-primary outcomes:

1. The first co-primary outcome is the healthfulness of participants' selections, operationalized as the average Ofcom Nutrient Profiling Model score of the products the participants select in the shopping task.
2. The second co-primary outcome is the carbon footprint of participants' selections, operationalized as the average carbon footprint (in CO_2-equivalents) of the products the participants select in the shopping task.

Secondary Outcomes

The secondary outcomes are:

- Selection outcomes:
  3. Calorie density, kcal per 100g
  4. Sugar density, g per 100g
  5. Sodium density, mg per 100g
  6. Saturated fat density, g per 100g
  7. Fiber density, g per 100g
  8. Protein density, g per 100g
  9. Total spending, USD ($)
- Psychological outcomes
  10. Thinking about health
  11. Thinking about climate impact
  12. Thinking about taste
  13. Negative emotions while shopping
  14. Positive emotions while shopping
  15. Injunctive norms to buy healthy foods
  16. Descriptive norms to buy healthy foods
  17. Injunctive norms to buy foods with low climate impact
  18. Descriptive norms to buy foods with low climate impact

Other Outcomes

The other outcomes are:

19. Perceived helpfulness of nutrition labels
20. Perceived helpfulness of climate labels
21. Perceived helpfulness of health swap recommendations
22. Perceived helpfulness of climate swap recommendations
23. Liking of nutrition labels
24. Liking of climate labels
25. Liking of health swap recommendations
26. Liking of climate swap recommendations
27. Acceptability of nutrition labels
28. Acceptability of climate labels
29. Acceptability of health swap recommendations
30. Acceptability of climate swap recommendations

Statistical Methods

1. We will describe participant characteristics by trial arm. We will use means and standard deviations to characterize continuous variables. We will use frequencies and percentages

to characterize categorical variables. We will also describe the number of swaps offered and proportion of swaps accepted in the intervention arms.

2. Analyses of the co-primary outcomes:
   a. We will use mixed effects linear regression to evaluate the effects of the interventions on the healthfulness and carbon footprint of participants' selections. In separate models for healthfulness and carbon footprint, we will regress the outcomes on indicator variables for trial arm (excluding the control arm as the reference category), an indicator variable for time period (baseline vs. follow-up) and the interaction between trial arm and time period. We will treat the intercept as random to account for repeated measures within participants. The treatment effects are given by the coefficients on the interaction terms. We will test whether each intervention arm (i.e., Health Swaps, Climate Swaps, or Combined Health + Climate Swaps) affects healthfulness and carbon footprint of participants' selections compared to the control arm. We will also test whether the effects of the 3 intervention arms on the healthfulness and carbon footprint of participants' selections differ from one another using Wald tests.
   b. We will test whether the effects of the interventions on the healthfulness and carbon footprint of participants' selections are moderated by age group (young adult ages 18-25 vs. older adult ages 26 and older), health consciousness (average responses to 4 items adapted from prior studies,[1,2] treated continuously), and environmental consciousness (average responses to the GREEN scale,[3] treated continuously). To test for moderation, we will add to the primary model indicators for the moderator, interactions between the moderator and trial arm, interactions between the moderator and time period (baseline vs. follow-up), and three-way interactions (i.e., difference-in-difference-in-differences) between the moderator, trial arm, and time period. We will use separate models for each co-primary outcome (healthfulness and carbon footprint) and moderator. We will test the joint significance of the difference-in-difference-in-differences interaction terms. If the primary analyses do not reveal differences between the three intervention arms, we may collapse across these arms for the moderation analyses.
   c. We will test whether the effects of the intervention on the co-primary outcomes differ between first and second exposure using mixed effects linear regression. Similar to the main model, we will regress the outcome on indicator variables for trial arm (excluding the control as the referent), week of data collection (i.e., week 2 or week 3, excluding week 1 [the baseline week] as the referent category), and the interactions between trial arm and week of data collection. We will use the model to estimate effects of the interventions on the outcomes during week 2 (vs. baseline) and week 3 (vs. baseline) and to test whether these effects differed from one another. If the analyses described above do not reveal differences between the three intervention arms, we may collapse across these arms for this analysis.

3. Analyses of secondary outcomes:
    a. We will use a similar mixed effects regression approach to evaluate the effect of the interventions on the secondary outcomes (e.g., calorie density, thinking about health).
4. Analyses of other outcomes
    a. We will descriptively report participants' responses to the perceived helpfulness, liking, and acceptability outcomes.

Sample Size Needs

We used G*Power to estimate sample size needs.[4] We estimated sample size needs to detect an effect of each intervention (health, climate, and combined) vs. the control of d=.15. This effect is considered small[69] and is conservative based on prior studies.[31,70,71] Assuming a two-tailed critical alpha of 0.05 and a correlation among repeated measures of 0.4, a sample size of 904 (226 per group) would yield 90% power to detect an effect of this size or larger. To account for potential missing data, we aimed to recruit a sample of 1,200 (300 per group).

Exclusions

In week 1, we will exclude participants who do not complete the survey. These participants will not be randomized and will not be invited to complete the remaining weeks of the study.

References

1. Rahamat S, Jeong E, Arendt SW, Xu Y. Menu labeling influence on purchase behaviors: Applying the theory of planned behavior and health consciousness. *Appetite*. 2022;172:105967. doi:10.1016/j.appet.2022.105967

2. Hearty ÁP, McCarthy SN, Kearney JM, Gibney MJ. Relationship between attitudes towards healthy eating and dietary behaviour, lifestyle and demographic factors in a representative sample of Irish adults. *Appetite*. 2007;48(1):1-11. doi:10.1016/j.appet.2006.03.329

3. Haws KL, Winterich KP, Naylor RW. Seeing the world through GREEN-tinted glasses: Green consumption values and responses to environmentally friendly products. *Journal of Consumer Psychology*. 2014;24(3):336-354. doi:10.1016/j.jcps.2013.11.002

4. Faul F, Erdfelder E, Lang AG, Buchner A. G*Power 3: A flexible statistical power analysis program for the social, behavioral, and biomedical sciences. *Behavior Research Methods*. 2007;39(2):175-191. doi:10.3758/BF03193146

5. Cohen J. *Statistical Power Analysis for the Behavioral Sciences*. Academic press; 2013.

6. Koutoukidis DA, Jebb SA, Ordóñez-Mena JM, et al. Prominent positioning and food swaps are effective interventions to reduce the saturated fat content of the shopping basket in an experimental online supermarket: a randomized controlled trial. *International Journal of Behavioral Nutrition and Physical Activity*. 2019;16(1):50. doi:10.1186/s12966-019-0810-9

7. Jansen L, van Kleef E, Van Loo EJ. The use of food swaps to encourage healthier online food choices: a randomized controlled trial. *International Journal of Behavioral Nutrition and Physical Activity*. 2021;18(1):156. doi:10.1186/s12966-021-01222-8

8. Schruff-Lim EM, Van Loo EJ, van der Lans IA, van Trijp HCM. Impact of food swap recommendations on dietary choices in an online supermarket: A randomized controlled trial. *Appetite*. 2024;194:107158. doi:10.1016/j.appet.2023.107158
