# Supplementary material for: Effect of food swap recommendations on the healthfulness and carbon footprint of grocery purchases in a naturalistic online store: A randomized parallel trial
Source: PLoS Med. 2026 Aug 21;23(8):e1004847. doi: 10.1371/journal.pmed.1004847 (PMC13497253; doi:10.1371/journal.pmed.1004847)
Supplement: Supplementary file 2 [file pmed.1004847.s001.docx]

**S1 Table.** Products shown in the naturalistic online store used in the trial

| **Food category** | **Subcategories** | **Description of included products** | **# products displayed** | **Correlation between carbon footprint and healthfulness^a^** |
| --- | --- | --- | --- | --- |
| Beverages | Coffee, tea & cocoa | Pre-packaged coffees and teas, tea bags tea, coffee pods, hot chocolate mix, coffee creamers, flavored syrups, cider, instant coffee | 774 | -0.07 |
|  | Juice | 100% fruit and vegetable juice, juice blends |  |  |
|  | Powdered drinks | Powdered drink mixes, diet powdered drink mixes, water enhancers |  |  |
|  | Soda | Sodas, diet sodas |  |  |
|  | Sports Drinks | Sports drinks, diet sports drinks, energy drinks, diet energy drinks |  |  |
|  | Water | Plain bottled water, seltzer water, flavored water, coconut water |  |  |
| Boxed and frozen meals | Convenience meals | Dinner kits and sides, macaroni and cheese, ready-to-heat meals, taco seasoning, taco shell kits and tortillas | 960 | 0.04 |
|  | Frozen dinners & entrees | Frozen pasta, frozen pot pies, frozen macaroni and cheese, frozen chicken nuggets, frozen patties, frozen meals |  |  |
|  | Frozen meat, chicken & seafood | Frozen chicken, frozen turkey, frozen fish sticks, frozen breaded fish |  |  |
|  | Frozen pizza, sandwiches & snacks | Frozen pizza, frozen burritos, frozen sandwiches |  |  |
|  | Frozen potatoes, fries & onion rings | Frozen tater tots, frozen French fries, frozen hashbrowns |  |  |
|  | Frozen breads & doughs | Frozen bagels, frozen bread, frozen biscuits, frozen dough, frozen garlic bread, frozen rolls |  |  |
| Milk and dairy products | Dairy alternatives | Plant-based milks | 881 | -0.54 |
|  | Milk and cream | Milk (may include some plant-based milks) |  |  |
|  | Cheese | Milk- and plant-based cheeses |  |  |
|  | Yogurt | Milk- and plant-based yogurts |  |  |
|  | Dairy alternatives | Plant-based milks and milkshakes |  |  |
| Meat and meat alternatives | Bacon, hot dogs, & sausages | Bacon, hotdogs, and sausages | 358 | -0.13 |
|  | Beef | Ground beef, patties, steaks, roasts, ribs |  |  |
|  | Chicken & turkey | Chicken breasts and thighs, ground chicken, ground turkey, turkey steaks, turkey sausage |  |  |
|  | Frozen meat & chicken | Chicken nuggets, plant-based beef, plant-based chicken nuggets, plant-based sausages, plant-based hot dogs, meatballs, pre-made burgers, other prepared meat products |  |  |
|  | Other meats | Bison, lamb, veal, venison |  |  |
|  | Packaged meat & sides | Pepperoni, turkey pepperoni, Lunchables and other snack packs, prepared or ready-to-heat potato sides, lunch meats (bologna, ham, turkey), jerky, some meat sauces |  |  |
|  | Pork | Pork roast, pork chops, pork ribs, ham steaks, pork tenderloin |  |  |
| Sweets and snacks | Snacks, chips, crackers & nuts | Crackers, dips and salsas, fruit snacks, meat snacks and jerky, nuts, seeds, popcorn, potato chips, pretzels, rice cakes, snack mixes, tortilla chips | 2,208 | -0.39 |
|  | Applesauce & fruit | Applesauce, canned fruit, fruit cups, fruit pouches, dried fruit (raisins, cranberries, apricots, mango, etc.) (note, does not include fresh fruit) |  |  |
|  | Candy | Chocolate, fruity candy, gummies, hard candies, gum, mints, |  |  |
|  | Cookies | Cookies |  |  |
| Soups | Soups | Broth, bouillon, condensed soup, ramen, ready-to-serve soup, soup mixes | 369 | -0.11 |

^a^Table reports the Pearson correlation coefficient between carbon footprint (kg of carbon dioxide equivalents per kg) and healthfulness (Ofcom score, ranging from 1 to 100).
