# Supplementary material for: Effect of food swap recommendations on the healthfulness and carbon footprint of grocery purchases in a naturalistic online store: A randomized parallel trial
Source: PLoS Med. 2026 Aug 21;23(8):e1004847. doi: 10.1371/journal.pmed.1004847 (PMC13497253; doi:10.1371/journal.pmed.1004847)
Supplement: Supplementary file 3 [file pmed.1004847.s002.docx]

**S2 Table.** Departments, categories, and subcategories used in the naturalistic online store used in the trial

| **Department** | **Category** | **Subcategory** |
| --- | --- | --- |
| Bakery | Cakes & Desserts | Cakes |
| Bakery | Cakes & Desserts | Cookies |
| Bakery | Cakes & Desserts | Cupcakes |
| Bakery | Cakes & Desserts | Other |
| Bakery | Cakes & Desserts | Other Desserts |
| Bakery | Cakes & Desserts | Pies |
| Bakery | Cakes & Desserts | Rolls |
| Bakery | Fresh Breads & Pastries | Bagels |
| Bakery | Fresh Breads & Pastries | Bread |
| Bakery | Fresh Breads & Pastries | Breakfast Pastries |
| Bakery | Fresh Breads & Pastries | Donuts |
| Bakery | Fresh Breads & Pastries | Muffins |
| Bakery | Fresh Breads & Pastries | Other |
| Bakery | Fresh Breads & Pastries | Rolls |
| Bakery | Gluten Free & Special Diets | Bagels |
| Bakery | Gluten Free & Special Diets | Cakes |
| Bakery | Gluten Free & Special Diets | Cookies |
| Bakery | Gluten Free & Special Diets | Cupcakes |
| Bakery | Gluten Free & Special Diets | Other |
| Bakery | Gluten Free & Special Diets | Other Desserts |
| Bakery | Gluten Free & Special Diets | Pies |
| Bakery | Special Occasion | Cookies |
| Bakery | Special Occasion | Other |
| Bakery | Special Occasion | Rolls |
| Beverages | Coffee, Tea & Cocoa | Coffee |
| Beverages | Coffee, Tea & Cocoa | Coffee Creamers |
| Beverages | Coffee, Tea & Cocoa | Coffee Drinks |
| Beverages | Coffee, Tea & Cocoa | Energy Drinks & Shots |
| Beverages | Coffee, Tea & Cocoa | Flavored Syrups & Mixes |
| Beverages | Coffee, Tea & Cocoa | Hot Cocoa & Cider |
| Beverages | Coffee, Tea & Cocoa | Iced Tea |
| Beverages | Coffee, Tea & Cocoa | Instant Coffee |
| Beverages | Coffee, Tea & Cocoa | Other |
| Beverages | Coffee, Tea & Cocoa | Tea Bags |
| Beverages | Juice | Other |
| Beverages | Liquor & Mixes | Other |
| Beverages | Liquor & Mixes | Pre-Made Cocktails & Mixes |
| Beverages | Powdered Drinks | Other |
| Beverages | Powdered Drinks | Tea Bags |
| Beverages | Soda | Other |
| Beverages | Soda | Pre-Made Cocktails & Mixes |
| Beverages | Soda | Seltzer Water |
| Beverages | Soda | Sparkling & Mineral Water |
| Beverages | Sports Drinks | Energy Drinks & Shots |
| Beverages | Sports Drinks | Other |
| Beverages | Sports Drinks | Sports Drinks |
| Beverages | Water | Bottled Water |
| Beverages | Water | Enhanced Water |
| Beverages | Water | Other |
| Beverages | Water | Sparkling & Mineral Water |
| Dairy | Butter | Other |
| Dairy | Cheese | Bar Cheese |
| Dairy | Cheese | Cottage Cheese |
| Dairy | Cheese | Cream Cheese |
| Dairy | Cheese | Other |
| Dairy | Cheese | Other Cheese |
| Dairy | Cheese | Processed Cheese |
| Dairy | Cheese | Ricotta |
| Dairy | Cheese | Shredded Cheese |
| Dairy | Cheese | Sliced Cheese |
| Dairy | Cheese | Snack Cheese |
| Dairy | Dairy Alternatives | Other |
| Dairy | Egg Substitutes & More | Other |
| Dairy | Eggs | Other |
| Dairy | Juice | Other |
| Dairy | Milk & Cream | Half & Half |
| Dairy | Milk & Cream | Heavy, Light & Whipping Cream |
| Dairy | Milk & Cream | Milk |
| Dairy | Milk & Cream | Non Dairy Creamers |
| Dairy | Milk & Cream | Other |
| Dairy | Pudding, Gelatin & Whipped Cream | Gelatin |
| Dairy | Pudding, Gelatin & Whipped Cream | Other |
| Dairy | Pudding, Gelatin & Whipped Cream | Pudding |
| Dairy | Pudding, Gelatin & Whipped Cream | Puddings & Gelatins |
| Dairy | Pudding, Gelatin & Whipped Cream | Whipped Cream |
| Dairy | Refrigerated Dough | Biscuit Dough & Pie Crusts |
| Dairy | Refrigerated Dough | Cookie Dough |
| Dairy | Refrigerated Dough | Other |
| Dairy | Sour Cream & Dips | Fresh Dips |
| Dairy | Sour Cream & Dips | Other |
| Dairy | Sour Cream & Dips | Sour Cream |
| Dairy | Yogurt | Drinkable Yogurt |
| Dairy | Yogurt | Multi Pack Yogurt |
| Dairy | Yogurt | Other |
| Dairy | Yogurt | Other Yogurt |
| Deli | Deli Meat & Cheese | Bologna & Other Deli Meat |
| Deli | Deli Meat & Cheese | Deli Cheese |
| Deli | Deli Meat & Cheese | Deli Meat Beef |
| Deli | Deli Meat & Cheese | Deli Meat Ham |
| Deli | Deli Meat & Cheese | Deli Meat Turkey & Chicken |
| Deli | Deli Meat & Cheese | Hot Dogs |
| Deli | Deli Meat & Cheese | Italian Deli Meat |
| Deli | Deli Meat & Cheese | Other |
| Deli | Deli Meat & Cheese | Other Deli Meat |
| Deli | Fine Cheese | Blue Cheeses |
| Deli | Fine Cheese | Bologna & Other Deli Meat |
| Deli | Fine Cheese | Bries & French Cheeses |
| Deli | Fine Cheese | Cheddars & Jacks |
| Deli | Fine Cheese | Cheese Spreads & More |
| Deli | Fine Cheese | Feta & Goat Cheeses |
| Deli | Fine Cheese | Gouda, Havarti & Other European Cheeses |
| Deli | Fine Cheese | Other |
| Deli | Fine Cheese | Other Cheese |
| Deli | Fine Cheese | Parmesan, Mozzarella & Other Italian Cheeses |
| Deli | Hummus, Dips & Snacks | Chips, Crackers & Accompaniments |
| Deli | Hummus, Dips & Snacks | Hummus & Dips |
| Deli | Hummus, Dips & Snacks | Other |
| Deli | Olives | Other |
| Deli | Special Occasion | Other |
| Frozen | Frozen Breads & Dough | Frozen Bread & Bagels |
| Frozen | Frozen Breads & Dough | Frozen Dough, Biscuits & Rolls |
| Frozen | Frozen Breads & Dough | Frozen Garlic Bread & Toast |
| Frozen | Frozen Breads & Dough | Other |
| Frozen | Frozen Desserts & Toppings | Frozen Bread & Bagels |
| Frozen | Frozen Desserts & Toppings | Frozen Cakes |
| Frozen | Frozen Desserts & Toppings | Frozen Dessert Toppings |
| Frozen | Frozen Desserts & Toppings | Frozen Pies, Crust & Pastry Dough |
| Frozen | Frozen Desserts & Toppings | Other |
| Frozen | Frozen Dinners & Entrees | Frozen Bagged Meals |
| Frozen | Frozen Dinners & Entrees | Frozen Entrees |
| Frozen | Frozen Dinners & Entrees | Frozen Nutritional Dinners |
| Frozen | Frozen Dinners & Entrees | Frozen Pasta |
| Frozen | Frozen Dinners & Entrees | Frozen Pot Pies |
| Frozen | Frozen Dinners & Entrees | Frozen Sandwiches |
| Frozen | Frozen Dinners & Entrees | Other |
| Frozen | Frozen Fruit | Frozen Novelties |
| Frozen | Frozen Fruit | Other |
| Frozen | Frozen Meat, Chicken & Seafood | Frozen Entrees |
| Frozen | Frozen Meat, Chicken & Seafood | Frozen Nutritional Dinners |
| Frozen | Frozen Meat, Chicken & Seafood | Frozen Sandwiches |
| Frozen | Frozen Meat, Chicken & Seafood | Frozen Seafood |
| Frozen | Frozen Meat, Chicken & Seafood | Other |
| Frozen | Frozen Pizza, Sandwiches & Snacks | Frozen Bread & Bagels |
| Frozen | Frozen Pizza, Sandwiches & Snacks | Frozen Pizza |
| Frozen | Frozen Pizza, Sandwiches & Snacks | Frozen Sandwiches |
| Frozen | Frozen Pizza, Sandwiches & Snacks | Other |
| Frozen | Frozen Potatoes, Fries & Onion Rings | Frozen Nutritional Dinners |
| Frozen | Frozen Potatoes, Fries & Onion Rings | Frozen Vegetables |
| Frozen | Frozen Potatoes, Fries & Onion Rings | Other |
| Frozen | Frozen Vegetables | Frozen Nutritional Dinners |
| Frozen | Frozen Vegetables | Frozen Vegetables |
| Frozen | Frozen Vegetables | Frozen Vegetables with Sauce |
| Frozen | Frozen Vegetables | Other |
| Frozen | Ice Cream & Novelties | Freeze Pops |
| Frozen | Ice Cream & Novelties | Frozen Novelties |
| Frozen | Ice Cream & Novelties | Frozen Pies, Crust & Pastry Dough |
| Frozen | Ice Cream & Novelties | Ice Cream |
| Frozen | Ice Cream & Novelties | Ice Cream Cakes |
| Frozen | Ice Cream & Novelties | Ice Cream Toppings |
| Frozen | Ice Cream & Novelties | Other |
| Frozen | Other Frozen | Other |
| Grocery | Applesauce & Fruit | Applesauce, Fruit Cups & Pouches |
| Grocery | Applesauce & Fruit | Canned Fruit |
| Grocery | Applesauce & Fruit | Dried Fruit |
| Grocery | Applesauce & Fruit | Other |
| Grocery | Baking Needs | Baking Mixes |
| Grocery | Baking Needs | Baking Soda, Powder, Starch & Yeast |
| Grocery | Baking Needs | Bread & Muffin Mixes |
| Grocery | Baking Needs | Bread Crumbs & Stuffings |
| Grocery | Baking Needs | Breakfast Bars & Granola |
| Grocery | Baking Needs | Canned & Dry Milk |
| Grocery | Baking Needs | Cooking Oils |
| Grocery | Baking Needs | Cooking Sprays & Shortening |
| Grocery | Baking Needs | Extracts & Colorings |
| Grocery | Baking Needs | Flour, Cornmeal & More |
| Grocery | Baking Needs | Frosting & Decorating |
| Grocery | Baking Needs | Marshmallows |
| Grocery | Baking Needs | Morsels, Nuts, Chocolate & Cocoa |
| Grocery | Baking Needs | Nut Butters & Other Spreads |
| Grocery | Baking Needs | Other |
| Grocery | Baking Needs | Other Baking |
| Grocery | Baking Needs | Pie Crusts & Fillings |
| Grocery | Baking Needs | Puddings & Gelatins |
| Grocery | Baking Needs | Sugar & Sweeteners |
| Grocery | Breakfast & Cereal | Breakfast Bars & Granola |
| Grocery | Breakfast & Cereal | Breakfast Drinks |
| Grocery | Breakfast & Cereal | Cereal |
| Grocery | Breakfast & Cereal | Oatmeal & Hot Cereal |
| Grocery | Breakfast & Cereal | Other |
| Grocery | Breakfast & Cereal | Other Baking |
| Grocery | Breakfast & Cereal | Pancakes, Waffles & Syrups |
| Grocery | Breakfast & Cereal | Toaster Pastries |
| Grocery | Candy | Candy |
| Grocery | Candy | Gum |
| Grocery | Candy | Large Candy Bars |
| Grocery | Candy | Nuts & Seeds |
| Grocery | Candy | Other |
| Grocery | Candy | Summer Candy |
| Grocery | Canned Meat & Seafood | Canned Clams, Oysters & More |
| Grocery | Canned Meat & Seafood | Canned Meats |
| Grocery | Canned Meat & Seafood | Canned Salmon |
| Grocery | Canned Meat & Seafood | Canned Shrimp & Crabmeat |
| Grocery | Canned Meat & Seafood | Other |
| Grocery | Canned Meat & Seafood | Sardines |
| Grocery | Canned Meat & Seafood | Stews & Chilis |
| Grocery | Canned Meat & Seafood | Tuna |
| Grocery | Canned Vegetables | Canned Beans |
| Grocery | Canned Vegetables | Canned Carrots, Potatoes & Spinach |
| Grocery | Canned Vegetables | Canned Tomatoes |
| Grocery | Canned Vegetables | Dried Beans |
| Grocery | Canned Vegetables | Other |
| Grocery | Canned Vegetables | Other Canned Vegetables |
| Grocery | Condiments & Dressing | BBQ Sauce |
| Grocery | Condiments & Dressing | Cooking Wine & Vinegar |
| Grocery | Condiments & Dressing | Croutons & Salad Toppings |
| Grocery | Condiments & Dressing | Gravy |
| Grocery | Condiments & Dressing | Hot Sauce |
| Grocery | Condiments & Dressing | Ketchup & Mustard |
| Grocery | Condiments & Dressing | Marinades & Cooking Sauces |
| Grocery | Condiments & Dressing | Mayonnaise |
| Grocery | Condiments & Dressing | Olives |
| Grocery | Condiments & Dressing | Organic & Natural Salad Dressing |
| Grocery | Condiments & Dressing | Other |
| Grocery | Condiments & Dressing | Other Condiments & Sauces |
| Grocery | Condiments & Dressing | Pickles & Peppers |
| Grocery | Condiments & Dressing | Relish |
| Grocery | Condiments & Dressing | Salad Dressing |
| Grocery | Condiments & Dressing | Spice Rubs |
| Grocery | Condiments & Dressing | Steak & Worcestershire Sauce |
| Grocery | Condiments & Dressing | Taco Seasoning |
| Grocery | Condiments & Dressing | Tartar & Seafood Sauces |
| Grocery | Convenience Meals | Dinner Kits & Sides |
| Grocery | Convenience Meals | Dips & Salsas |
| Grocery | Convenience Meals | Macaroni & Cheese |
| Grocery | Convenience Meals | Other |
| Grocery | Convenience Meals | Ready to Heat Meals |
| Grocery | Convenience Meals | Rice Side Dishes |
| Grocery | Convenience Meals | Spices |
| Grocery | Convenience Meals | Taco Seasoning |
| Grocery | Convenience Meals | Taco Shell Kits & Tortillas |
| Grocery | Cookies | Crackers |
| Grocery | Cookies | Other |
| Grocery | Cookies | Snack Size & Multi Pack |
| Grocery | Dairy Alternatives | Other |
| Grocery | International | Baking Mixes |
| Grocery | International | Bread Crumbs & Stuffings |
| Grocery | International | Bread, Wraps & Tortillas |
| Grocery | International | Breakfast Bars & Granola |
| Grocery | International | Candy |
| Grocery | International | Canned & Dry Milk |
| Grocery | International | Cereal |
| Grocery | International | Crackers |
| Grocery | International | Dips & Salsas |
| Grocery | International | Flour, Cornmeal & More |
| Grocery | International | Nuts & Seeds |
| Grocery | International | Oatmeal & Hot Cereal |
| Grocery | International | Other |
| Grocery | International | Other Baking |
| Grocery | International | Pancakes, Waffles & Syrups |
| Grocery | International | Pasta |
| Grocery | International | Pasta Sauces |
| Grocery | International | Pickles & Peppers |
| Grocery | International | Potato Chips |
| Grocery | International | Pretzels |
| Grocery | International | Ready to Heat Meals |
| Grocery | International | Rice |
| Grocery | International | Rice Cakes |
| Grocery | International | Snack Size & Multi Pack |
| Grocery | International | Taco Shell Kits & Tortillas |
| Grocery | International | Tortilla Chips |
| Grocery | Packaged Bread, Muffins & Pastries | Bagels |
| Grocery | Packaged Bread, Muffins & Pastries | Bread, Wraps & Tortillas |
| Grocery | Packaged Bread, Muffins & Pastries | Breakfast Bars & Granola |
| Grocery | Packaged Bread, Muffins & Pastries | Buns & Rolls |
| Grocery | Packaged Bread, Muffins & Pastries | English Muffins |
| Grocery | Packaged Bread, Muffins & Pastries | Hamburger & Hot Dog Buns |
| Grocery | Packaged Bread, Muffins & Pastries | Other |
| Grocery | Packaged Bread, Muffins & Pastries | Snack Cakes & Pastries |
| Grocery | Pasta, Sauces & Sides | Morsels, Nuts, Chocolate & Cocoa |
| Grocery | Pasta, Sauces & Sides | Other |
| Grocery | Pasta, Sauces & Sides | Pasta |
| Grocery | Pasta, Sauces & Sides | Pasta Sauces |
| Grocery | Peanut Butter, Jelly & Honey | Honey |
| Grocery | Peanut Butter, Jelly & Honey | Jam, Jelly & Preserves |
| Grocery | Peanut Butter, Jelly & Honey | Nut Butters & Other Spreads |
| Grocery | Peanut Butter, Jelly & Honey | Other |
| Grocery | Peanut Butter, Jelly & Honey | Peanut Butter |
| Grocery | Rice, Beans & Potatoes | Canned Beans |
| Grocery | Rice, Beans & Potatoes | Dinner Kits & Sides |
| Grocery | Rice, Beans & Potatoes | Dried Beans |
| Grocery | Rice, Beans & Potatoes | Other |
| Grocery | Rice, Beans & Potatoes | Potato Side Dishes |
| Grocery | Rice, Beans & Potatoes | Rice |
| Grocery | Rice, Beans & Potatoes | Rice Side Dishes |
| Grocery | Sauces, Spices & Seasonings | BBQ Sauce |
| Grocery | Sauces, Spices & Seasonings | Extracts & Colorings |
| Grocery | Sauces, Spices & Seasonings | Marinades & Cooking Sauces |
| Grocery | Sauces, Spices & Seasonings | Other |
| Grocery | Sauces, Spices & Seasonings | Other Condiments & Sauces |
| Grocery | Sauces, Spices & Seasonings | Salt |
| Grocery | Sauces, Spices & Seasonings | Spice Rubs |
| Grocery | Sauces, Spices & Seasonings | Spices |
| Grocery | Snacks, Chips, Crackers & Nuts | Baking Mixes |
| Grocery | Snacks, Chips, Crackers & Nuts | Breakfast Bars & Granola |
| Grocery | Snacks, Chips, Crackers & Nuts | Crackers |
| Grocery | Snacks, Chips, Crackers & Nuts | Dips & Salsas |
| Grocery | Snacks, Chips, Crackers & Nuts | Fruit Snacks |
| Grocery | Snacks, Chips, Crackers & Nuts | Meat Snacks & Jerky |
| Grocery | Snacks, Chips, Crackers & Nuts | Nuts |
| Grocery | Snacks, Chips, Crackers & Nuts | Nuts & Seeds |
| Grocery | Snacks, Chips, Crackers & Nuts | Other |
| Grocery | Snacks, Chips, Crackers & Nuts | Popcorn |
| Grocery | Snacks, Chips, Crackers & Nuts | Potato Chips |
| Grocery | Snacks, Chips, Crackers & Nuts | Pretzels |
| Grocery | Snacks, Chips, Crackers & Nuts | Rice Cakes |
| Grocery | Snacks, Chips, Crackers & Nuts | Snack Size & Multi Pack |
| Grocery | Snacks, Chips, Crackers & Nuts | Snacks & Party Mixes |
| Grocery | Snacks, Chips, Crackers & Nuts | Taco Shell Kits & Tortillas |
| Grocery | Snacks, Chips, Crackers & Nuts | Tortilla Chips |
| Grocery | Soups | Broth & Boullion |
| Grocery | Soups | Condensed Soup |
| Grocery | Soups | Organic & Natural |
| Grocery | Soups | Other |
| Grocery | Soups | Ramen & Noodles |
| Grocery | Soups | Ready To Serve Soup |
| Grocery | Soups | Rice |
| Grocery | Soups | Soup Mixes |
| Grocery | Soups | Stews & Chilis |
| Meat | Bacon, Hot Dogs & Sausage | Bacon |
| Meat | Bacon, Hot Dogs & Sausage | Frozen Sausage |
| Meat | Bacon, Hot Dogs & Sausage | Hot Dogs |
| Meat | Bacon, Hot Dogs & Sausage | Other |
| Meat | Bacon, Hot Dogs & Sausage | Sausage |
| Meat | Beef | All Natural |
| Meat | Beef | Burger Patties & Ground Beef |
| Meat | Beef | Chuck Steaks & Roasts |
| Meat | Beef | Cube & Stew Beef |
| Meat | Beef | Frozen Chicken & Turkey |
| Meat | Beef | Frozen Meatballs |
| Meat | Beef | Frozen Sandwich Meat & More |
| Meat | Beef | Frozen Sausage |
| Meat | Beef | Loin Steaks & Roasts |
| Meat | Beef | Other |
| Meat | Beef | Other Beef |
| Meat | Beef | Ribeye Steaks & Rib Roasts |
| Meat | Beef | Round Steaks & Roasts |
| Meat | Beef | Veal |
| Meat | Chicken & Turkey | Buffalo & Wild Game Meats |
| Meat | Chicken & Turkey | Chicken |
| Meat | Chicken & Turkey | Marinated Meat |
| Meat | Chicken & Turkey | Other |
| Meat | Chicken & Turkey | Turkey |
| Meat | Frozen Meat & Chicken | Frozen Beef Patties |
| Meat | Frozen Meat & Chicken | Frozen Chicken & Turkey |
| Meat | Frozen Meat & Chicken | Frozen Meatballs |
| Meat | Frozen Meat & Chicken | Frozen Sandwich Meat & More |
| Meat | Frozen Meat & Chicken | Other |
| Meat | Marinated & Seasoned Meat | Burger Patties & Ground Beef |
| Meat | Marinated & Seasoned Meat | Chicken |
| Meat | Marinated & Seasoned Meat | Marinated Meat |
| Meat | Marinated & Seasoned Meat | Other |
| Meat | Marinated & Seasoned Meat | Other Pork |
| Meat | Marinated & Seasoned Meat | Sausage |
| Meat | Marinated & Seasoned Meat | Seasoned Meat & More |
| Meat | Other Meats | Buffalo & Wild Game Meats |
| Meat | Other Meats | Lamb |
| Meat | Other Meats | Other |
| Meat | Other Meats | Other Beef |
| Meat | Packaged Meat & Sides | Entrees & Sides |
| Meat | Packaged Meat & Sides | Lunch Packs |
| Meat | Packaged Meat & Sides | Meat Pies |
| Meat | Packaged Meat & Sides | Other |
| Meat | Packaged Meat & Sides | Packaged Lunchmeat |
| Meat | Packaged Meat & Sides | Sausage |
| Meat | Pork | All Natural |
| Meat | Pork | Ham Steaks |
| Meat | Pork | Marinated Meat |
| Meat | Pork | Other |
| Meat | Pork | Packaged Meats |
| Meat | Pork | Pork Butts & Ribs |
| Meat | Pork | Pork Chops |
| Meat | Pork | Pork Tenderloin & Roasts |
| Meat | Pork | Smoked Ham |
