# Supplementary material for: Effect of food swap recommendations on the healthfulness and carbon footprint of grocery purchases in a naturalistic online store: A randomized parallel trial
Source: PLoS Med. 2026 Aug 21;23(8):e1004847. doi: 10.1371/journal.pmed.1004847 (PMC13497253; doi:10.1371/journal.pmed.1004847)
Supplement: Supplementary file 4 [file pmed.1004847.s003.docx]

**S3 Table.** Survey measures used in trial

| **Construct** | **Item**  **[programming notes]** | **Response scale** | **Visit** | | |
| --- | --- | --- | --- | --- | --- |
|  |  |  | **1** | **2** | **3** |
|  | **Eligibility** |  |  |  |  |
| Age | How old are you? | **[force response]**  [free response, #, restricted whole numbers to 0-115]  **[if <18, skip to study termination]** | X |  |  |
| Country | Where do you currently live? | **[force response]**  1=United States  2=United Kingdom  3=Somewhere else  **[if not 1, skip to study termination]** | X |  |  |
|  | **Commitment Check – Visit 1**  **[display for visit 1 only]** |  |  |  |  |
| Study instructions | This is a **3-part study.** You will be asked to complete **3 online surveys** – 1 survey per week for 3 weeks. This is the first survey, which you should complete today. We will contact you again in about 1 week to complete the second survey, and about 1 week after that to complete the third survey.  The quality of our research depends on you completing all 3 parts of the study.  Do you commit to completing all 3 parts of the study? | **[randomize order of responses]**  1=I can’t promise either way  2=Yes, I will  3=No, I will not  **[cannot proceed until 2 is selected]** | X |  |  |
| Commitment to provide thoughtful answers | We care about the quality of our data. For us to get the most accurate measures of your opinions, it is important that you provide thoughtful answers to each question in the survey.  Do you commit to providing thoughtful answers to the questions in the survey? | **[randomize order of responses]**  1=I can’t promise either way  2=Yes, I will  3=No, I will not  **[cannot proceed until 2 is selected]** | X |  |  |
|  | **Commitment Check – Visit 2**  **[display for visit 2 only]** |  |  |  |  |
| Study instructions | Remember, this is a **3-part study** in which you will complete **3 online surveys** – 1 survey per week for 3 weeks. You completed the first survey last week. You can complete the second survey today. We will contact you again in about 1 week to complete the third survey.  The quality of our study depends on you completing all 3 parts of the study.  Do you commit to completing the remaining 2 parts of the study?  **[page break]** | **[randomize order of responses]**  1=I can’t promise either way  2=Yes, I will  3=No, I will not  **[cannot proceed until 2 is selected]** |  | X |  |
| Commitment to provide thoughtful answers | We care about the quality of our data. For us to get the most accurate measures of your opinions, it is important that you provide thoughtful answers to each question in the survey.  Do you commit to providing thoughtful answers to the questions in the survey? | **[randomize order of responses]**  1=I can’t promise either way  2=Yes, I will  3=No, I will not  **[cannot proceed until 2 is selected]** |  | X |  |
|  | **Commitment Check – Visit 3**  **[display for visit 3 only]** |  |  |  |  |
| Commitment to thoughtful answers | We care about the quality of our data. For us to get the most accurate measures of your opinions, it is important that you provide thoughtful answers to each question in the survey.  Do you commit to providing thoughtful answers to the questions in the survey? | **[randomize order of responses]**  1=I can’t promise either way  2=Yes, I will  3=No, I will not  **[cannot proceed until 2 is selected]** |  |  | X |
|  | **Prompt Visit 1**  **[display for visit 1 only]** |  |  |  |  |
| Prompt visit 1 | **Welcome to the Online Grocery Shopping Study.**  In this study, you will view an online grocery store and complete a shopping task. Then, you will answer survey questions. |  | X |  |  |
|  | **Prompt Visit 2**  **[display for visits 2 and 3 only]** |  |  |  |  |
| Prompt visits 2 and 3 | **Welcome back to the Online Grocery Shopping Study.**  Similar to the survey you took last week, you will view an online grocery store and complete a shopping task. Then, you will answer survey questions. |  |  | X | X |
|  | **Introduction to Shopping Task** |  |  |  |  |
| Prompt | First, you will view an online grocery store and complete a shopping task. The online grocery store looks like a typical store, but only offers the following types of foods:   - Beverages - Dairy & alternatives - Meats & alternatives - Boxed & frozen meals - Soups - Sweet & salty snacks   Enter the online store and select the items you would like to purchase among the types of foods offered in this store. You should shop as you normally would for these types of foods. For example, if you usually buy milk but not soup, you should select your preferred milk from the store, but you don’t need to select a soup. Your budget is $50.  You will not be asked to spend your own money. However, you should make your selections carefully because **we will choose 25 participants to receive the groceries they selected during 1 of their study visits, plus any money left over in their budget.** If you are chosen to receive your selections, they will be delivered to an address of your choosing. This means you should select products you actually want. |  | X | X | X |
| Shopping task instructions | We care about the quality of our data. For us to get the most accurate information, it is important that you understand these instructions.  Please select the answer option that is correct based on the information you have read above:  **[page break]** | **[randomize order of responses]**  1=I have to pay for the items I select with my own money  2=There is a chance I will be chosen to receive the groceries I selected  3=No participants in this study will receive the groceries they selected  **[cannot proceed until 2 is selected]** | X | X | X |
| More shopping task instructions | You will now enter the online grocery store. The store can take up to 20 seconds to load on a fast internet connection and a bit longer on a slower internet connection. Thank you for your patience.  As a reminder, the grocery store looks like a typical store, but only offers certain types of foods including beverages, dairy & alternatives, meat & alternatives, soups, boxed & frozen meals, and sweet & salty snacks.  Once you enter the store, please shop as you normally would for these types of foods. Your budget is $50. If you have questions while you shop, click the "help" button at the top right side of the online store.  Click the “Next Page” button to view the link to the store. |  | X | X | X |
|  | **Shopping Task Link** |  |  |  |  |
| Store re-direct link | Below is the link to access the online grocery store.  The store works best in modern internet browsers (Chrome, Firefox, Safari, Edge). Please allow up to 20 seconds for the store to load. If the store does not load after 20 seconds, you can try refreshing the page.  If the store does not load after refreshing, please try using the link in a different browser.  **{taskurl}**  If you encounter technical difficulties loading the store, reports on issues (errors shown, screenshots, description of problem, etc.) are highly appreciated in order to improve the grocery store platform. Reports can be sent to [blinded].  **[participants complete shopping task before being redirected back to the rest of the study]** |  | X | X | X |
|  | **Introduction to Rest of Survey** |  |  |  |  |
| Prompt | **Thank you for shopping in the online store. You will now answer some questions.** |  | X | X | X |
|  | **Reactions to Labels** |  |  |  |  |
| Shopping task prompt | **These next questions are about your experience shopping in the online store.** |  | X | X | X |
| Noticing Nutrition Facts Labels | When you were selecting foods and beverages in the shopping task, did you see any Nutrition Facts Panel labels? The Nutrition Facts Panel looks like this.  **[Display screenshot of Nutrition Facts Label]** | [display vertically]  1=Yes  0=No  2=Not sure | X | X | X |
| Noticing nutrition labels | While you were shopping, did you notice any other nutrition-related labels next to the products? (other than the Nutrition Facts Panel) | 1=Yes  0=No  2=Not sure | X | X | X |
| Noticing climate labels | While you were shopping, did you notice any climate-related labels next to the products? | 1=Yes  0=No  2=Not sure | X | X | X |
|  | **Reactions to Swaps** |  |  |  |  |
| Noticing health swaps | While you were shopping, did you notice any recommendations to switch to a product with a better nutrition grade?  **[page break]** | 1=Yes  0=No  2=Not sure | X | X | X |
| Self-reported swap acceptance | **[show if Noticing health swaps = 1]**  Did you accept any of the recommendations to switch to a product with a better nutrition grade? | 1=Yes  0=No  2=Not sure | X | X | X |
| Reasons for accepting health swap | **[show if Noticing health swaps = 1]**  Why or why not?  **[page break]** | [free response text box] | X | X | X |
| Noticing climate swaps | While you were shopping, did you notice any recommendations to switch to a product with a better climate grade?  **[page break]** | 1=Yes  0=No  2=Not sure | X | X | X |
| Self-reported swap acceptance | **[show if Noticing climate swaps = 1]**  Did you accept any of the recommendations to switch to a product with a better climate grade? | 1=Yes  0=No  2=Not sure | X | X | X |
| Reasons for accepting climate swap | **[show if Noticing climate swaps = 1]**  Why or why not? | [free response text box] | X | X | X |
|  | **Emotional Reactions** |  |  |  |  |
| Affective reactions – stem | **[format affective reactions as a matrix; randomize order of emotions within the matrix]**  While you were shopping, how much did you feel… | 1=Not at all  2=A little  3=Somewhat  4=Very  5=Extremely | X | X | X |
| Negative affective reaction - guilty | guilty? |  | X | X | X |
| Negative affective reaction – ashamed | ashamed? |  | X | X | X |
| Negative affective reaction – worry | worried? |  | X | X | X |
| Positive affective reaction- inspired | inspired? |  | X | X | X |
| Positive affective reaction- proud | proud? |  | X | X | X |
| Positive affective reaction – reassured | reassured? |  | X | X | X |
|  | **Elaboration** |  |  |  |  |
| Elaboration-stem | We are interested in how you selected products while you were grocery shopping. When you selected foods, how much did you think about each of the following characteristics?  **[format items below as a matrix; randomize order of attributes]** | 1=Not at all  2=Very little  3=Somewhat  4=Quite a bit  5=A great deal | X | X | X |
| Elaboration-Health | Health |  | X | X | X |
| Elaboration-Taste | Taste |  | X | X | X |
| Elaboration-Climate impact | Climate impact |  | X | X | X |
|  | **Norms** |  |  |  |  |
| Prompt | **Say how much you agree or disagree with the next statements.** |  | X | X | X |
| Injunctive norms - health | People who are important to me think I should buy healthy foods. | 1=Strongly disagree  2=Somewhat disagree  3=Neither disagree nor agree  4=Somewhat agree  5=Strongly agree | X | X | X |
| Injunctive norms - climate | People who are important to me think I should buy foods with low climate impact. | 1=Strongly disagree  2=Somewhat disagree  3=Neither disagree nor agree  4=Somewhat agree  5=Strongly agree | X | X | X |
| Descriptive norms - health | Most shoppers buy healthy foods when they are shopping for groceries. | 1=Strongly disagree  2=Somewhat disagree  3=Neither disagree nor agree  4=Somewhat agree  5=Strongly agree | X | X | X |
| Descriptive norms – climate impact | Most shoppers buy foods with low climate impact when they are shopping for groceries. | 1=Strongly disagree  2=Somewhat disagree  3=Neither disagree nor agree  4=Somewhat agree  5=Strongly agree | X | X | X |
|  | **Label and Swap Acceptability** |  |  |  |  |
| Prompt – health labels | Food companies could place nutrition labels on the foods they sell, like the labels below. Labels with “A” or “B” scores mean the food is healthier, while those with “D” or “F” scores are less healthy. You might have seen these labels earlier in the study.  Look closely at the labels. Then, answer the questions below.   |  |  |  | X |
| Acceptability – helpfulness | These labels would help me choose healthier foods. | 1=Not at all  2=A little  3=Somewhat  4=Quite a bit  5=A great deal |  |  | X |
| Acceptability – liking | I like these labels. | 1=Not at all  2=A little  3=Somewhat  4=Quite a bit  5=A great deal |  |  | X |
| Acceptability – approval | How much would you approve or disapprove of these labels being displayed on foods sold in grocery stores?  **[page break]** | 1=Strongly disapprove  2=Somewhat disapprove  3=Neither disapprove nor approve  4=Somewhat approve  5=Strongly approve |  |  | X |
| Prompt – climate labels | Food companies could place climate labels on the foods they sell, like the labels below. Labels with “A” or “B” scores mean the food has a lower climate impact, while those with “D” or “F” scores have a worse climate impact. You might have seen these labels earlier in the study.  Look closely at the labels. Then, answer the questions below.   |  |  |  | X |
| Acceptability – helpfulness | These labels would help me choose foods with lower climate impact. | 1=Not at all  2=A little  3=Somewhat  4=Quite a bit  5=A great deal |  |  | X |
| Acceptability – liking | I like these labels. | 1=Not at all  2=A little  3=Somewhat  4=Quite a bit  5=A great deal |  |  | X |
| Acceptability – approval | How much would you approve or disapprove of these labels being displayed on foods sold in grocery stores?  **[page break]** | 1=Strongly disapprove  2=Somewhat disapprove  3=Neither disapprove nor approve  4=Somewhat approve  5=Strongly approve |  |  |  |
| Prompt – Health swaps | Grocery stores could provide shoppers who select less healthy foods with a recommendation for similar foods that are healthier, like in the image below. You might have seen this type of recommendation earlier in the survey.  Look closely at this recommendation. Then, answer the questions below.  **[display image of swap] [not shown here to preserve copyright]** |  |  |  | X |
| Acceptability – helpfulness | This type of recommendation would help me choose healthier foods. | 1=Not at all  2=A little  3=Somewhat  4=Quite a bit  5=A great deal |  |  | X |
| Acceptability – liking | I like these recommendations. | 1=Not at all  2=A little  3=Somewhat  4=Quite a bit  5=A great deal |  |  | X |
| Acceptability – approval | How much would you approve or disapprove of these recommendations being offered by grocery stores?  **[page break]** | 1=Strongly disapprove  2=Somewhat disapprove  3=Neither disapprove nor approve  4=Somewhat approve  5=Strongly approve |  |  |  |
| Prompt – Climate swaps | Grocery stores could provide shoppers who select foods that have higher climate impact with a recommendation for similar foods that have lower climate impact, like in the image below. You might have seen this type of recommendation earlier in the survey.  Look closely at this recommendation. Then, answer the questions below.  **[display image of swap] [not shown here to preserve copyright]** |  |  |  | X |
| Acceptability – helpfulness | This type of recommendation would help me choose foods with lower climate impact. | 1=Not at all  2=A little  3=Somewhat  4=Quite a bit  5=A great deal |  |  | X |
| Acceptability – liking | I like these recommendations. | 1=Not at all  2=A little  3=Somewhat  4=Quite a bit  5=A great deal |  |  | X |
| Acceptability – approval | How much would you approve or disapprove of these recommendations being offered by grocery stores? | 1=Strongly disapprove  2=Somewhat disapprove  3=Neither disapprove nor approve  4=Somewhat approve  5=Strongly approve |  |  | X |
|  | **Store Acceptability and Validity** |  |  |  |  |
| Prompt | **The next questions are about the online grocery store.** |  | X |  |  |
| Overall ease of using this store | Overall, how difficult or easy was it to use this online grocery store? | 1=Very difficult  2=Difficult  3=Neither difficult nor easy  4=Easy  5=Very easy | X |  |  |
| Prompt | **Say how much you agree or disagree with the following statements.** |  | X |  |  |
| Likelihood of shopping in online store again | If I had the chance, I would shop in this online store again. | 1=Strongly disagree  2=Somewhat disagree  3=Neither disagree nor agree  4=Somewhat agree  5=Strongly agree | X |  |  |
| Would choose similar products in the real-world | If I completed this shopping task in the real world, I would pick products similar to the products I chose in this study. | 1=Strongly disagree  2=Somewhat disagree  3=Neither disagree nor agree  4=Somewhat agree  5=Strongly agree | X |  |  |
| Choices were similar to usual grocery purchases | The foods I selected in this online grocery store were similar to my usual grocery purchases. | 1=Strongly disagree  2=Somewhat disagree  3=Neither disagree nor agree  4=Somewhat agree  5=Strongly agree | X |  |  |
| Store felt like a real store | This online grocery store felt like a real online grocery store. | 1=Strongly disagree  2=Somewhat disagree  3=Neither disagree nor agree  4=Somewhat agree  5=Strongly agree | X |  |  |
|  | **Health and Environmental Consciousness** |  |  |  |  |
| Prompt | **Say how much you agree or disagree with the following statements.** |  | X |  |  |
| Health consciousness | I am someone who is concerned with the long-term health effects of my food choices. | 1=Strongly disagree  2=Somewhat disagree  3=Neither disagree nor agree  4=Somewhat agree  5=Strongly agree | X |  |  |
| Health consciousness | I am someone who carefully considers the health consequences of my food choices. | 1=Strongly disagree  2=Somewhat disagree  3=Neither disagree nor agree  4=Somewhat agree  5=Strongly agree | X |  |  |
| Health consciousness | I reflect about my health a lot. | 1=Strongly disagree  2=Somewhat disagree  3=Neither disagree nor agree  4=Somewhat agree  5=Strongly agree | X |  |  |
| Health consciousness | I make conscious efforts to try and eat a healthy diet.  **[page break]** | 1=Strongly disagree  2=Somewhat disagree  3=Neither disagree nor agree  4=Somewhat agree  5=Strongly agree | X |  |  |
| Prompt | **Say how much you agree or disagree with the statements below.** |  | X |  |  |
| GREEN scale (environmental consciousness) | It is important to me that the products I use do not harm the environment. | 1=Strongly disagree  2=Somewhat disagree  3=Neither disagree nor agree  4=Somewhat agree  5=Strongly agree | X |  |  |
| GREEN scale (environmental consciousness) | I consider the potential environmental impact of my actions when making many of my decisions. | 1=Strongly disagree  2=Somewhat disagree  3=Neither disagree nor agree  4=Somewhat agree  5=Strongly agree | X |  |  |
| GREEN scale (environmental consciousness) | My purchase habits are affected by my concern for the environment. | 1=Strongly disagree  2=Somewhat disagree  3=Neither disagree nor agree  4=Somewhat agree  5=Strongly agree | X |  |  |
| GREEN scale (environmental consciousness) | I am concerned about wasting the resources of our planet. | 1=Strongly disagree  2=Somewhat disagree  3=Neither disagree nor agree  4=Somewhat agree  5=Strongly agree | X |  |  |
| GREEN scale (environmental consciousness) | I would describe myself as environmentally responsible. | 1=Strongly disagree  2=Somewhat disagree  3=Neither disagree nor agree  4=Somewhat agree  5=Strongly agree | X |  |  |
|  | **Demographics** |  |  |  |  |
| Introduction to demographics | **We are asking the questions in the next section to better understand who completed our survey.** |  | X |  |  |
| Gender | How do you identify? | 1=Woman  2=Man  3=Non-binary  4=Prefer to self-describe:_____ | X |  |  |
| Hispanic ethnicity | Are you of Hispanic, Latino, or Spanish origin? | 1=Yes  0=No | X |  |  |
| Race | What is your race? (Check all that apply). | [Check all that apply]  1=American Indian or Alaska Native  2=Asian  3=Black or African American  4=Native Hawaiian or Other Pacific Islander  5=White  6=Another race:________ | X |  |  |
| Education | What is the highest degree or level of school you have completed?  **[page break]** | 1=Less than high school  2=High school graduate (or GED)  3=Some college or technical school  4=Associate’s degree  5=Bachelor’s degree  6=Graduate or professional degree | X |  |  |
| Nutrition Label Use | How often do you use the Nutrition Facts Panel label when deciding to buy packaged foods? | 1=Never  2=Rarely  3=Sometimes  4=Most of the time  5=Always | X |  |  |
| Shopping frequency at Hannaford stores | In the past year, how often have you bought groceries from Hannaford supermarkets? | 1=Never  2=Rarely  3=Sometimes  4=Often  5=Very often | X |  |  |
| Online grocery amount | **In the past year, about what percentage of your groceries did you buy online?** This includes any groceries you buy through the internet or a mobile app for delivery or pick-up, including from Amazon Fresh, Walmart.com, Instacart, Target.com or your local grocery store.  **[page break]** | 1=Less than 25%  2=25-49%  3=50-74%  4=75% or more | X |  |  |
| Party identification | Do you consider yourself a Democrat, a Republican, an Independent, or something else? | 1=Strong Democrat  2=Moderate Democrat  3=Lean Democrat  4=Independent  5=Lean Republican  6=Moderate Republican  7=Strong Republican  8=Something else:____ | X |  |  |
| Household size | How many people are in your household, including you? | [# of people [restricted to 1-20, whole numbers] | X |  |  |
| Children | How many children (ages 0-18) currently live in your household? | ____ [#, restricted to 0-15, whole numbers] | X |  |  |
| Income | Which of the following categories best describes your total household income before taxes in the last 12 months? | 1=Less than $10,000  2=$10,000 to $14,999  3=$15,000 to $24,999  4=$25,000 to $34,999  5=$35,000 to $49,999  6=$50,000 to $74,999  7=$75,000 to $99,999  8=$100,000 to $149,999  9=$150,000 to $199,999  10=$200,000 or more | X |  |  |
|  | **Closure** |  |  |  |  |
| Anything else | Is there anything else you want to tell us? | [free response] | X | X | X |
|  | **Debrief** |  |  |  |  |
| Debrief | Thank you for taking part in this research study. There was some information about the study that we did not share with you at the beginning of your participation, so that you would react honestly. We would now like to fully inform you about the nature of this research and explain a bit more about the survey you just completed. In the consent form and survey, we indicated that 1 in 50 participants would be selected to receive the groceries they selected from the online grocery store shipped directly to their address in addition to the amount agreed upon when they entered this survey. In actuality, these participants will instead receive $50 distributed to their PayPal account (the budget for the shopping task) in addition to the amount agreed upon when you entered this survey.  We stated that you might receive the groceries you selected because we wanted you to behave as you normally would when choosing foods and to choose options you would actually want to purchase. This study was conducted by researchers at Stanford University who are interested in the effects of food swaps, or suggestions to choose healthier or more environmentally sustainable food or beverage options. We will use this study to determine how effective different types of food swaps are at changing which products people choose.  Please feel free to contact the investigator, Anna Grummon, with any questions or if you have any concerns about your participation. She can be reached via email at agrummon@stanford.edu. If you would like a copy of this debriefing form, please save this page to your computer. |  |  |  | X |
