# Supplementary material for: Effect of food swap recommendations on the healthfulness and carbon footprint of grocery purchases in a naturalistic online store: A randomized parallel trial
Source: PLoS Med. 2026 Aug 21;23(8):e1004847. doi: 10.1371/journal.pmed.1004847 (PMC13497253; doi:10.1371/journal.pmed.1004847)
Supplement: Supplementary file 5 [file pmed.1004847.s004.docx]

**S4 Table.** Comparison of characteristics of the study sample (*n*=1,201 US adults) to national estimates

|  | **Study sample**  **%** | **National estimate**  **%** |
| --- | --- | --- |
| Age |  |  |
| 18-25 years | 50% | 13% |
| 26-39 years | 29% | 24% |
| 40-59 years | 17% | 32% |
| 60 years or older | 4% | 31% |
| Gender |  |  |
| Woman | 53% | 51% |
| Man | 45% | 49% |
| Non-binary or another gender | 1% | NA |
| Latino(a) or Hispanic Ethnicity | 8% | 18% |
| Race |  |  |
| American Indian or Alaska Native | 1% | 1% |
| Asian, Native Hawaiian, or Pacific Islander | 5% | 6% |
| Black or African American | 31% | 12% |
| White | 59% | 63% |
| Another race or multiracial | 4% | 18% |
| Education |  |  |
| High school diploma or less | 11% | 37% |
| Some college or associate’s degree | 16% | 29% |
| College graduate | 46% | 21% |
| Graduate degree | 27% | 13% |
| Household income, annual |  |  |
| $0 to $49,999 | 28% | 30% |
| $50,000 to $99,999 | 40% | 27% |
| $100,000 to $149,999 | 20% | 17% |
| $150,000 or more | 12% | 26% |

^a^National estimates for age, gender, race and ethnicity, and education are survey-weighted estimates among adults (ages 18 years and older) in the 2023 American Community Survey (ACS) 1-year Public Use Microdata Sample (PUMS).^[1]^ National estimate of % of people identifying as non-binary or another gender is listed as “NA” because the ACS did not include an option for people to identify as non-binary or another gender. National estimates for income are from the Current Population Survey, 2024.^[2]^

1. United States Census Bureau. 2023 PUMS Data. Census.gov. September 19, 2024. Accessed December 16, 2024. https://www.census.gov/programs-surveys/acs/microdata/access/2023.html
2. United States Census Bureau UC. HINC-01. Selected Characteristics of Households by Total Money Income. Census.gov. August 8, 2025. Accessed September 12, 2025. https://www.census.gov/data/tables/time-series/demo/income-poverty/cps-hinc/hinc-01.html
