# Supplementary material for: Effect of food swap recommendations on the healthfulness and carbon footprint of grocery purchases in a naturalistic online store: A randomized parallel trial
Source: PLoS Med. 2026 Aug 21;23(8):e1004847. doi: 10.1371/journal.pmed.1004847 (PMC13497253; doi:10.1371/journal.pmed.1004847)
Supplement: Supplementary file 6 [file pmed.1004847.s005.docx]

**S5 Table.** Effects of the climate, health, and climate + health swaps on the healthfulness and carbon footprint of food and beverage purchases, by food group, *n*=1,201 US adults

| **Outcome** | **Climate swaps** | | **Health swaps** | | **Climate + health swaps** | |
| --- | --- | --- | --- | --- | --- | --- |
|  | B | (95% CI) | B | (95% CI) | B | (95% CI) |
| **Healthfulness (Ofcom score, 1–100)** |  |  |  |  |  |  |
| Beverages | 0.3 | (-0.3, 0.8) | **1.6** | **(1.0, 2.2)** | **0.8** | **(0.2, 1.4)** |
| Boxed and frozen meals | 1.9 | (-0.03, 3.8) | **5.2** | **(3.3, 7.1)** | **4.7** | **(2.9, 6.6)** |
| Meat and meat alternatives | **5.8** | **(2.2, 9.3)** | **7.1** | **(3.5, 10.7)** | **6.4** | **(2.8, 10.0)** |
| Milk and dairy products | 1.3 | (-1.3, 3.8) | **6.1** | **(3.6, 8.7)** | 2.3 | (-0.2, 4.9) |
| Soups | 0.3 | (-0.8, 1.4) | 0.9 | (-0.2, 2.0) | **1.6** | **(0.5, 2.7)** |
| Sweets and snacks | **3.9** | **(1.1, 6.8)** | **10.5** | **(7.7, 13.4)** | **8.2** | **(5.4, 11.1)** |
| **Carbon footprint, kg CO_2_-eq per kg** |  |  |  |  |  |  |
| Beverages | -0.4 | (-0.8, 0.04) | 0.0 | (-0.4, 0.5) | -0.4 | (-0.8, 0.1) |
| Boxed and frozen meals | **-1.4** | **(-2.3, -0.6)** | 0.6 | (-0.2, 1.5) | -0.7 | (-1.5, 0.1) |
| Meat and meat alternatives | **-9.6** | **(-15.5, -3.7)** | 2.3 | (-3.6, 8.3) | **-9.9** | **(-15.9, -4.0)** |
| Milk and dairy products | **-1.4** | **(-2.1, -0.7)** | **-1.0** | **(-1.7, -0.2)** | -0.7 | (-1.4, 0.05) |
| Soups | **-2.7** | **(-4.3, -1.0)** | -0.01 | (-1.6, 1.6) | **-2.4** | **(-4.0, -0.8)** |
| Sweets and snacks | **-4.2** | **(-6.0, -2.4)** | **-3.3** | **(-5.1, -1.5)** | **-2.8** | **(-4.6, -1.1)** |

*Abbreviations*. CI, confidence interval; CO_2_-eq, carbon dioxide equivalents.

*Note*. Table shows the impact of each swaps intervention compared to control (no intervention), given as the difference in changes from baseline to follow-up between the swaps arm and the control arm (B) along with the corresponding 95% CI. Table shows effects pooled across the first and second exposure to the swaps. Estimates are from linear mixed models, regressing the outcome on indicator variables for each trial arm, study visit, the interaction between trial arm and visit, and a random intercept to account for repeated measures within participants. **Bolded** rows indicate the difference is statistically significant, *p*<0.05.
