# Supplementary material for: Effect of food swap recommendations on the healthfulness and carbon footprint of grocery purchases in a naturalistic online store: A randomized parallel trial
Source: PLoS Med. 2026 Aug 21;23(8):e1004847. doi: 10.1371/journal.pmed.1004847 (PMC13497253; doi:10.1371/journal.pmed.1004847)
Supplement: Supplementary file 7 [file pmed.1004847.s006.docx]

**S1 Text.** Supplemental Methods

We applied labels within six food groups: beverages, boxed and frozen meals, milk and dairy products, meat and meat alternatives, soups, and sweet and salty snacks.

**Applying climate labels.** In the climate swaps and climate + health swaps groups, we divided products in each food category (i.e., beverages, meals, milk and dairy products, meat and meat alternatives, soups, and sweet and salty snacks) into quintiles based on the product’s carbon footprint per kg. We applied “A,” “B,” “C,” “D,” and “F” labels to the lowest, second lowest, middle, second highest, and highest quintiles of carbon footprints, respectively.

**Applying health labels.** In the health swaps and climate + health swaps groups, we applied “A” or “B” health labels to products with Ofcom scores meeting the UK’s regulatory cutoffs for products eligible to be marketed to children (62 for foods and 68 for beverages, after converting to 1–100 scores).^1,2^ Among the products that were at or above these regulatory cutoffs, we calculated the median Ofcom score of products in each food category (i.e., within beverages, meals, milk and dairy products, meat and meat alternatives, soups, and sweet and salty snacks). We then assigned “A” labels to products above that median and “B” labels to those below that median but above the regulatory cutoff. We then divided the remaining products (i.e., those below the regulatory cutoff) in each food category into tertiles based on their Ofcom scores and then applied “C,” “D,” and “F” labels to the top, middle, and bottom tertile of these remaining products. For milk and dairy products, we applied “A” and “B” labels separately for liquid dairy products (using the regulatory cutoff for beverages) and solid dairy products (using the regulatory cutoff for foods), then applied the “C,” “D,” and “F” products to the top, middle, and bottom tertile of all remaining dairy products combined (liquid or solid).

**Identifying swaps.** We identified swaps based on a product categorization system used in the grocery store from which products in our store were scraped. The categorization system sorted products into departments, categories, and subcategories (e.g., Lean Cuisine Cafe Classics Herb Chicken Breast is in the “Frozen” department, “Frozen Dinners & Entrees” category, and “Frozen Entrees” subcategory). The online grocery store presented swaps for selected products that had a “C,” “D,” or “F” climate or health label. The online grocery store identified all possible product swaps and presented an initial set of up to 8 potential product swaps, beginning with products that were in the same product subcategory and had an “A” or “B” climate or health label. Participants could choose to see more potential swaps, which were loaded in batches of up to 8. After all possible products swaps in the same subcategory were exhausted, product swaps in the same category were presented, followed by products in the same department. Swaps were presented in order from most to least healthful based on Ofcom scores in the health swaps arm, lowest to highest climate impact based on carbon footprint in the climate swaps arm, and by the combination of climate and health impact in the climate + health swaps arm. Specifically, products that received an “A” on both dimensions were shown first, products that received an “A” on one dimension and a “B” on the other were shown second, and products with a “B” on both dimensions were shown last. Within those groupings, we showed more healthful products first.

**Analytic approach.** In the primary analyses, we regressed the outcome on indicator variables for trial arm (i.e., climate swaps, health swaps, or climate + health swaps, excluding the control arm as the reference category), indicator variables for study visit (visit 2 or visit 3, excluding visit 1 [baseline visit] as the reference category), and interactions between the trial arms and study visit. We used the models to estimate whether the 3 swaps interventions led to changes in the two primary outcomes from baseline to the intervention period (estimated as the weighted average of effects at visit 2 and visit 3), net of changes in the control group and to test whether the intervention effects differed from one another. In this model, the coefficients on the interactions between the trial arms and study visit 2 (study visit 3) represent the effects of each intervention at study visit 2 (study visit 3) compared to baseline. To estimate pooled effects across the two intervention study visits, we calculated the weighted average of these coefficients, weighting by the number of observations at each study visit.

To test for moderation, we added indicator variables for the moderator and interaction terms between the moderator, trial arm, and study visit. The parameters of interest are the coefficients on the three-way interactions between the moderator, trial arm, and study visit, which represent the difference in the effect of the interventions by levels of the moderator.

To test whether the effect of the intervention differed between the first and second exposure to the intervention, we used the same model as for the primary analyses. We tested whether the coefficients on the interactions between the trial arms and study visit 2 differed from the coefficients on the interactions between the trial arms and study visit 3, i.e., whether the effects of each intervention differed between the first and second exposures.

**References**

1.	Rayner M, Scarborough P, Lobstein T. The UK Ofcom Nutrient Profiling Model: Defining “healthy” and “unhealthy” foods and drinks for TV advertising to children. 2009. Accessed June 12, 2022. https://docs.google.com/viewer?url=https%3A%2F%2Fwww.ndph.ox.ac.uk%2Fcpnp%2Ffiles%2Fabout%2Fuk-ofcom-nutrient-profile-model.pdf

2.	Rayner M. Nutrient profiling for regulatory purposes. *Proc Nutr Soc*. 2017;76(3):230-236. doi:10.1017/S0029665117000362
