# Supplementary material for: Effect of food swap recommendations on the healthfulness and carbon footprint of grocery purchases in a naturalistic online store: A randomized parallel trial
Source: PLoS Med. 2026 Aug 21;23(8):e1004847. doi: 10.1371/journal.pmed.1004847 (PMC13497253; doi:10.1371/journal.pmed.1004847)
Supplement: Supplementary file 8 [file pmed.1004847.s007.docx]

**S1 Figure.** Acceptability of the naturalistic online store used in the trial

*Note.* Figure shows the percentage of participants who somewhat or strongly agreed with each statement.
